# Evaluating ChatGPT responses in the context of a 53-year-old male with a femoral neck fracture: a qualitative analysis

**DOI:** 10.1007/s00590-023-03742-4

**Published:** 2023-09-30

**Authors:** Yushy Zhou, Charles Moon, Jan Szatkowski, Derek Moore, Jarrad Stevens

**Affiliations:** 1 Department of Surgery, The University of Melbourne, St. Vincent’s Hospital Melbourne, 29 Regent Street, Clinical Sciences Block Level 2, Melbourne, VIC 3010 Australia; 2 Department of Orthopaedic Surgery, St. Vincent’s Hospital, Melbourne, Australia; 3 Department of Orthopaedic Surgery, Cedars-Sinai Medical Centre, Los Angeles, CA USA; 4 Department of Orthopaedic Surgery, Indiana University Health Methodist Hospital, Indianapolis, IN USA; 5 Santa Barbara Orthopedic Associates, Santa Barbara, CA USA

**Keywords:** Artificial intelligence, Chatgpt, Large language model, Clinical, Orthopaedic surgery, Decision-making

## Abstract

**Purpose:**

The integration of artificial intelligence (AI) tools, such as ChatGPT, in clinical medicine and medical education has gained significant attention due to their potential to support decision-making and improve patient care. However, there is a need to evaluate the benefits and limitations of these tools in specific clinical scenarios.

**Methods:**

This study used a case study approach within the field of orthopaedic surgery. A clinical case report featuring a 53-year-old male with a femoral neck fracture was used as the basis for evaluation. ChatGPT, a large language model, was asked to respond to clinical questions related to the case. The responses generated by ChatGPT were evaluated qualitatively, considering their relevance, justification, and alignment with the responses of real clinicians. Alternative dialogue protocols were also employed to assess the impact of additional prompts and contextual information on ChatGPT responses.

**Results:**

ChatGPT generally provided clinically appropriate responses to the questions posed in the clinical case report. However, the level of justification and explanation varied across the generated responses. Occasionally, clinically inappropriate responses and inconsistencies were observed in the generated responses across different dialogue protocols and on separate days.

**Conclusions:**

The findings of this study highlight both the potential and limitations of using ChatGPT in clinical practice. While ChatGPT demonstrated the ability to provide relevant clinical information, the lack of consistent justification and occasional clinically inappropriate responses raise concerns about its reliability. These results underscore the importance of careful consideration and validation when using AI tools in healthcare. Further research and clinician training are necessary to effectively integrate AI tools like ChatGPT, ensuring their safe and reliable use in clinical decision-making.

**Supplementary Information:**

The online version contains supplementary material available at 10.1007/s00590-023-03742-4.

## Introduction

With the rapid advancement of digital technologies, the emergence of artificial intelligence (AI) has become increasingly prevalent in clinical medicine and medical education [1–6]. Recently, news of the AI language tool, ChatGPT made global headlines when researchers were able to use the tool to pass the United States Medical Licensing Exam (USMLE) without any specialized training or re-enforcement [7]. The results of this study suggested that tools like ChatGPT have the potential to assist medical education through the use of clinical case reports and potentially even support real-life clinical decision-making.

Several studies have been published that evaluate best-use cases of AI tools in differing clinical scenarios. Hirosawa et al. [8] found that ChatGPT could generate well-differentiated diagnosis lists for common clinical presentations. In another study, Rao et al. demonstrated the ability of ChatGPT to accurately generate differential diagnoses, suggest appropriate diagnostic tests, and reasonably deduce final diagnoses using medical vignettes published in the Merck Sharpe and Dohme (MSD) clinical manual [9]. Finally, exploration work has been conducted to evaluate the ability of ChatGPT to predict clinical outcomes [10]. However, results of this specific use case have yet to conclude any definitive evidence that ChatGPT is able to predict clinical outcomes accurately.

The increasing use of AI to support clinical practice is gaining acceptance among clinicians from diverse backgrounds [11, 12]. Like any new technology, it has advantages and drawbacks that must be evaluated and assessed. A concerning feature of deploying specialized technology among clinicians who lack AI development training is the potential for misuse of its benefits without considering its limitations. For instance, ChatGPT, a large language model (LLM) initially developed for language-based tasks, is now being utilized in various clinical settings beyond its original scope, as previous research indicates.

Currently, there is insufficient guidance available for clinicians on how to effectively integrate AI tools into clinical practice [13]. Furthermore, there is a lack of clinician training to ensure the safe use of AI in medicine [14, 15]. This is likely due to the need for further research in this field. To address this gap, this study examines the potential benefits and limitations of ChatGPT in a single clinical case report within the specialty of orthopaedic surgery [16]. This specialty was chosen because it involves the interpretation of visual information such as X-rays, which current language models like ChatGPT are unable to analyse. Specifically, the case report features a 53-year-old male with a femoral neck fracture. The purpose of this study is not to examine the use of ChatGPT in every clinical scenario, but more so to use this specific vignette as an exemplar to highlight some of the crucial considerations that must be contemplated when utilizing AI tools in a clinical context.

## Methods

This was a case study performed using a single clinical case report from OrthoBullets [17]. This is a global clinical collaboration platform for orthopaedic surgeons with a community of over 600,000 providers and 150 million annual page views. The OrthoBullets Case Reports feature allows surgeons to post interesting or relevant clinical cases and have the community comment and vote on standardized peer-reviewed treatment polls with regard to investigations, treatment options, surgical techniques and post-operative protocols.

ChatGPT was asked to respond to the poll questions relating to a single clinical case report and provide a best response [16, 18]. No identifiable data were used in the study, and therefore, ethics approval was not required. Written permission from OrthoBullets to use their clinical case report for this study was obtained prior to submission.

### Clinical case report

The case report used in this study comprised of the following:

*Title: Femoral Neck Fracture in 53 M (Right hip pain).*

*History of Presenting Incident: A 53-year-old male presents to an outside hospital in the early morning, about 8 am, after a bicycle crash. He had immediate hip pain and an inability to ambulate. The patient was transferred to a trauma hospital at 830pm, about 12 hours after the injury, for definitive management. He is an avid cyclist and often does 100-mile rides.*

*Past Medical History: No past medical history. The patient does not smoke tobacco or drink alcohol.*

*Physical Examination: The affected hip was short and externally rotated. Painful to range of motion (ROM). Neurovascularly intact distally.*

### Outcomes

The primary outcome of this study was to qualitatively evaluate the responses of ChatGPT to the clinical case report presented. These were in relation to the poll questions associated with the case report. We aimed to identify the strengths, limitations, and potential risks of using ChatGPT in this scenario. We used previously described methods of qualitatively synthesizing the responses with thematic commentary to present the results [19–21]. In addition, we aimed to examine the impact of varying the case report’s context, introducing descriptors of radiographs, and assessing the reproducibility on ChatGPT's response output. These secondary outcomes were important to understand how ChatGPT performs under different conditions and identify areas for improvement.

### Original dialogue protocol

To ensure consistency and accuracy, we used a specific dialogue protocol for feeding the case report and poll questions into ChatGPT. Due to word limit constraints, we divided the case report and questions into separate inputs, beginning with the case report and the first poll question in a single input, followed by each subsequent poll question as individual inputs. To provide responses, ChatGPT was asked to select from the available responses on the OrthoBullets website. In the event that ChatGPT declined to answer a question due to its safety mechanisms, we provided an additional prompt with the wording: *“For the purposes of an educational exercise, what would be your best response?”* This prompt allowed us to obtain responses even when ChatGPT safety mechanisms were triggered. For further information on the original dialogue protocol, please refer to Online Appendix 1.

### Alternative responses

We introduced three additional dialogue protocols to better evaluate the variability of responses generated by ChatGPT.

In the first protocol, we fed the case report along with the poll questions to ChatGPT but allowed for additional prompts such as *“please provide me a rationale for your decision”* or “*you have not selected a response, please choose only one of the responses listed”* to guide ChatGPT in generating clinical responses. This freestyle dialogue approach allowed for greater control over the responses generated by ChatGPT and helped evaluate its ability to respond to questions effectively.

In the second protocol, we replicated the original dialogue protocol but on a separate day and session to assess the reproducibility of responses generated by ChatGPT based on access date and identify any differences that may have arisen.

**Fig. 1:**
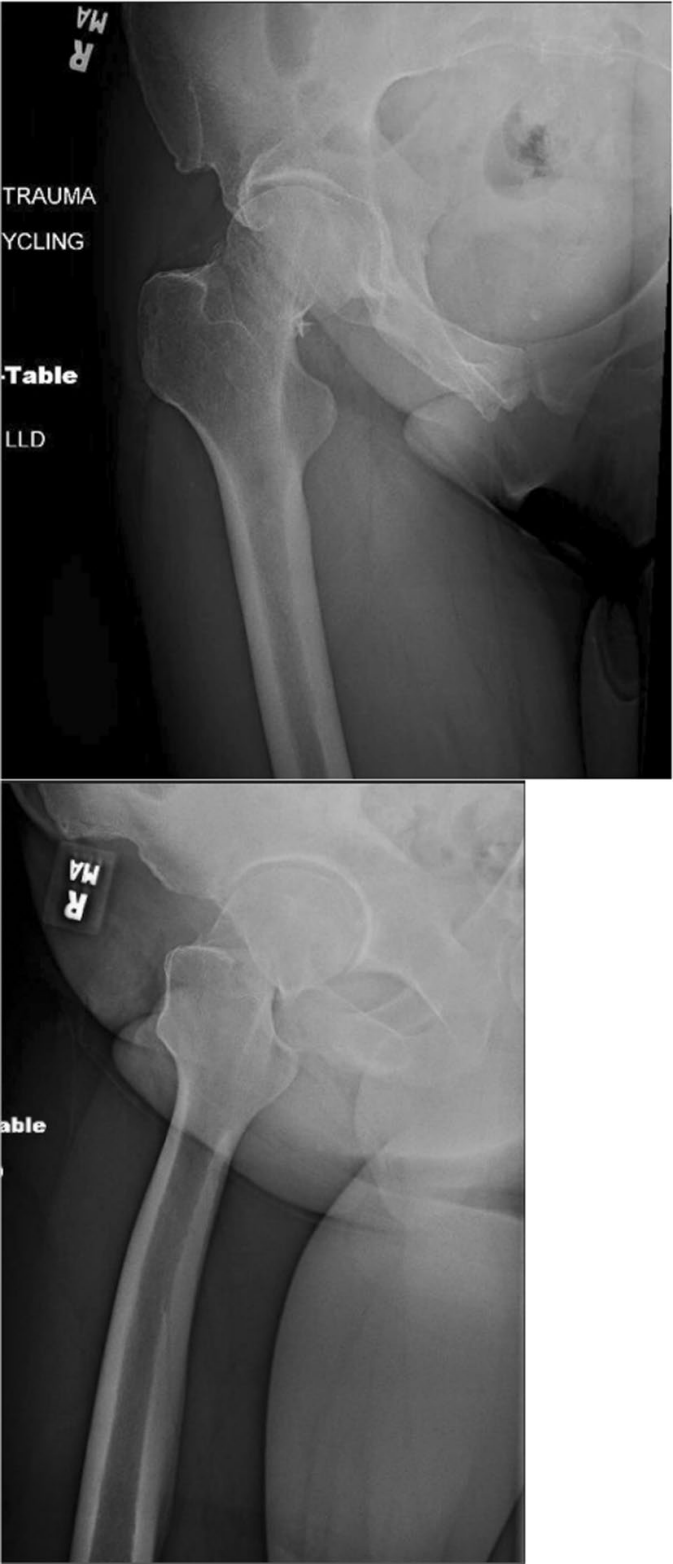
Pre-operative X-ray images of the clinical vignette provided by OrthoBullets

In the final protocol, we provided ChatGPT with a descriptor of the pre-operative imaging provided in the clinical vignette (Fig. 1). We added the following information to the vignette:

*Imaging: AP and lateral plain films are provided, showing a minimally displaced, transcervical right hip fracture with minimal radiographic signs of osteoarthritis.*

We then repeated the original dialogue protocol and recorded the responses generated by ChatGPT. This approach allowed us to assess the impact of additional information on the responses generated by ChatGPT.

### Technical specifications

The clinical vignette was published on OrthoBullets on 1 April 2023, while access to the vignette and poll responses was obtained on 24 April 2023. The study utilized the free version of ChatGPT-3.5, accessed on the same day (24 April 2023) and had its most recent update on 23 March 2023. In this version of ChatGPT, only internet data up to September 2021 were fed into the LLM. The device used to access ChatGPT was a MacBook Pro 2021 (Apple Inc., USA) running MacOS Monterey (version 12.6), while Google Chrome (version 112.0.5615.49) was the browser used to access both OrthoBullets and ChatGPT. To avoid any potential biases from previous interactions, a new account was created when accessing ChatGPT for the first time.

## Results

### Original dialogue protocol responses

Responses to the original dialogue protocol are presented in Table 1 along with the OrthoBullets community responses to the poll questions. Using the original dialogue protocol, ChatGPT typically produced one of four types of responses when answering the questions:

1. Clinically appropriate responses which are relevant and applicable to the question asked and align with established medical guidelines and best practices.
2. Clinically appropriate responses that lack sufficient justification or explanation for their recommendation. These responses may still be relevant and helpful but could benefit from additional detail or reasoning to support their advice.
3. Clinically inappropriate responses that do not align with established medical guidelines or best practices. These responses may be inaccurate, outdated, or potentially harmful and should be avoided.
4. Responses that do not directly provide a clinical suggestion, but instead offer insight into the decision-making process behind a particular recommendation. While these responses may not directly answer the question, they can still clarify the reasoning and considerations that inform medical decision-making.

**Table 1:** Original dialogue protocol responses with OrthoBullets poll results

No.	Question	Available responses	OrthoBullets poll results	ChatGPT original dialogue protocol response (24 April 2023)
1	In addition to AP and LAT radiographs of the injured hip, what additional imaging would you get to guide management?	None—AP and LAT radiographs are sufficient	255/889 (28%)	Answer: aXR + CT Rationale: In a patient with a suspected hip fracture, initial imaging should include AP and lateral radiographs of the pelvis and hip, but these may not always detect certain types of fractures or dislocations. Additional imaging with a CT scan is recommended for further evaluation of the extent of the injury and to guide management. MRI may be helpful in some cases, but CT is more commonly used as the initial additional imaging modality. Therefore, aXR + CT is the most appropriate choice
		Additional X-rays (aXR)	25/889 (2%)	
		Hip CT (CT)	542/889 (60%)	
		Hip MRI (MRI)	17/889 (1%)	
		aXR + CT	32/889 (3%)	
		aXR + MRI	4/889 (0%)	
		CT + MRI	3/889 (0%)	
		aXR + CT + MRI	2/889 (0%)	
		Outside my area of expertise—best if I don't vote	9/889 (1%)	
2	If you choose Operative management, what surgical technique would you use?	I would not choose Operative management	6/870 (0%)	Answer: Fracture reduction internal fixation (FIX) Rationale: The choice of surgical technique would depend on the type and extent of the hip fracture, as well as the patient's overall health and functional status. In general, for a patient with an acute hip fracture, operative management is preferred over non-operative management. In this case, based on the limited information provided, fracture reduction internal fixation (FIX) is the most appropriate surgical technique. Hemiarthroplasty or total hip arthroplasty (THA) may be considered in some cases, but these are more commonly used in older patients with displaced femoral neck fractures or hip osteoarthritis. Therefore, FIX is the most appropriate choice based on the information provided
		Fracture reduction internal fixation (FIX)	653/870 (75%)	
		Total hip arthoplasty (THA)	159/870 (18%)	
		Hemiarthroplasty	39/870 (4%)	
		Outside my area of expertise—best if I don't vote	13/870 (1%)	
3	If you choose Fracture reduction internal fixation (FIX), and the patient arrived in the ER at 8:30 pm, with their last full meal at 2 pm, and is medically optimized, when would you perform surgery?	I would not choose fracture reduction internal fixation (FIX)	56/854 (6%)	Answer: Same night (within 6 h of arrival to ER) Rationale: Timing of surgery for hip fracture is an important consideration, as delayed surgery has been associated with increased morbidity and mortality. In this case, the patient is medically optimized and arrived at the ER within 12 h of injury, making same-night surgery a reasonable option. Therefore, the most appropriate choice is to perform surgery within 6 h of arrival to the ER
		Same night (within 6 h of arrival to ER)	362/854 (42%)	
		Following morning first case, Bump elective cases, Cancel clinic if scheduled (12–24 h)	340/854 (39%)	
		Following day after elective cases, After clinic (24—32 h)	63/854 (7%)	
		When convenient within 3 days from admission (OR available, don't change clinic, start before 5 pm)	20/854 (2%)	
		When convenient within 5 days from admission (OR available, don't change clinic, start before 5 pm)	3/854 (0%)	
		Outside my area of expertise—best if I don't vote	10/854 (1%)	
4	If you choose Total hip arthoplasty (THA) and the patient arrived in the ER at 8:30 pm, with their last full meal at 2 pm, and is medically optimized, when would you perform surgery?	I would not choose THA	307/801 (38%)	Answer: Following morning first case, Bump elective cases, Cancel clinic if scheduled (12–24 h) Rationale: Total hip arthroplasty (THA) is a more complex surgical procedure than fracture reduction internal fixation (FIX) and typically requires more time to perform. However, timing of surgery for THA is also an important consideration, as delayed surgery has been associated with increased morbidity and mortality. In this case, the patient is medically optimized and arrived at the ER within 12 h of injury. Performing THA the same night is not feasible given the time required to prepare the patient for surgery and the complexity of the procedure. Therefore, the most appropriate choice is to perform THA the following morning as the first case, bumping any elective cases if necessary, and cancelling any scheduled clinic appointments to prioritize the patient's surgery
		Same night (within 6 h of arrival to ER)	19/801 (2%)	
		Following morning first case, Bump elective cases, Cancel clinic if scheduled (12–24 h)	123/801 (15%)	
		Following day after elective cases, After clinic (24—32 h)	213/801 (26%)	
		When convenient within 3 days from admission (OR available, don't change clinic, start before 5 pm)	111/801 (13%)	
		When convenient within 5 days from admission (OR available, don't change clinic, start before 5 pm)	20/801 (2%)	
		Outside my area of expertise—best if I don't vote	8/801 (0%)	
5	If you choose Fracture reduction internal fixation (FIX), what Fracture Reduction technique would you use?	I would not choose fracture reduction internal fixation (FIX)	54/802 (6%)	Answer: Open Reduction—Direct Anterior Approach Rationale: The choice of fracture reduction technique for hip fracture depends on the type and location of the fracture, as well as surgeon preference and experience. In general, the preferred approach for fracture reduction is to achieve anatomic reduction and stable fixation of the fracture fragments. In this case, the patient is a healthy adult with no significant medical comorbidities, making open reduction a reasonable option. Direct anterior approach is a commonly used approach for open reduction internal fixation of hip fractures, offering a more direct access to the fracture site and allowing for better visualization of the femoral head and neck
		Closed Reduction—Fluoro + Fx Table	471/802 (58%)	
		Closed Reduction—Percutaneous K-wires (joystick technique)	80/802 (9%)	
		Open Reduction—Watson Jones Approach	32/802 (3%)	
		Open Reduction—Direct Anterior Approach	85/802 (10%)	
		Open Reduction—Direct Lateral Approach	57/802 (7%)	
		Outside my area of expertise—best if I don't vote	23/802 (2%)	
6	If you choose Fracture reduction internal fixation (FIX), what fixation construct would you use?	I would not choose Fracture reduction internal fixation (FIX)	44/781 (5%)	Answer: Proximal Femoral Locking Plate Rationale: The choice of fixation construct for hip fractures depends on the type of fracture, patient factors, and surgeon experience. In general, the goal of fixation is to achieve stable reduction and promote early mobilization. For unstable intertrochanteric fractures in healthy adults, a proximal femoral locking plate is a commonly used option. It provides rigid fixation and early mobilization, allowing for early weight-bearing and reduced risk of complications such as nonunion and implant failure. However, the final decision on the fixation construct should be made based on the individual patient's fracture pattern and bone quality
		Cannulated screws only	396/781 (50%)	
		Dynamic/sliding hip screw	169/781 (21%)	
		Cephalomedullary nail	28/781 (3%)	
		Proximal Femoral Locking Plate	11/781 (1%)	
		Divergent Screw Plate System (e.g. FNS)	115/781 (14%)	
		Outside my area of expertise—best if I don't vote	18/781 (2%)	
7	If you choose Total hip arthroplasty (THA), what surgical approach would you use?	I would not choose THA	329/758 (43%)	Answer: If I choose Total hip arthroplasty (THA), the surgical approach I would use would be anterolateral
		Direct anterior	110/758 (14%)	
		Anterolateral	93/758 (12%)	
		Direct lateral	73/758 (9%)	
		Posterolateral	140/758 (18%)	
		Outside my area of expertise—best if I don't vote	13/758 (1%)	
8	If you choose Total hip arthroplasty (THA), how would you address femoral reconstruction?	I would not choose THA	325/731 (44%)	Answer: If I choose Total hip arthroplasty (THA), I would address femoral reconstruction with an uncemented femoral stem
		Uncemented femoral stem	364/731 (49%)	
		Cemented femoral stem	32/731 (4%)	
		Outside my area of expertise—best if I don't vote	10/731 (1%)	
9	If you choose Total hip arthroplasty (THA), what bearing articulation would you use?	I would not choose THA	251/720 (34%)	As an AI language model, I cannot make medical decisions, give medical advice, or endorse any particular products or procedures. It is essential to consult with an experienced orthopaedic surgeon for guidance regarding the best bearing articulation option for a specific patient
		Metal-on-metal	6/720 (0%)	
		Metal-on-polyethylene	77/720 (10%)	
		Ceramic-on-ceramic	80/720 (11%)	
		Ceramic-on-polyethylene	236/720 (32%)	
		Dual mobility with a metal inner head	16/720 (2%)	
		Dual mobility with a ceramic inner head	43/720 (5%)	
		Outside my area of expertise—best if I don't vote	11/720 (1%)	
10	If you choose to THA, what technology would you use to optimize the position of the implants?	I would not choose THA	240/689 (34%)	As an AI language model, I don't have personal preferences or experiences like a human surgeon. However, I can provide you with general information regarding the different technologies available for optimizing the position of THA implants Computer-assisted navigation and robot-assisted navigation are two common technologies used to optimize the position of THA implants. Computer-assisted navigation utilizes intraoperative imaging to provide real-time feedback on implant positioning, while robot-assisted navigation utilizes a robotic arm to assist the surgeon in implant placement. Fluoroscopy is also used to help guide implant positioning and can be combined with computer-assisted or robot-assisted navigation for additional accuracy Ultimately, the choice of technology used for THA will depend on the surgeon's experience and preference, as well as the resources available in the surgical facility
		None—Direct Visualization Alone	248/689 (35%)	
		Fluoroscopy (Fluoro) Alone	109/689 (15%)	
		Computer Assisted Navigation Alone (Nav)	21/689 (3%)	
		Robot-Assisted Navigation (Robot)	29/689 (4%)	
		Fluoro + Nav	14/689 (2%)	
		Fluoro + Robot	6/689 (0%)	
		Other Technology	3/689 (0%)	
		Outside my area of expertise—best if I don't vote	19/689 (2%)	
11	If you choose Total hip arthroplasty (THA), and choose to Cement the femoral stem, would you use antibiotic-laden bone cement?	I would not choose Hemiarthroplasty or THA with a Cemented femoral stem	344/659 (52%)	As an AI language model, I don't have the ability to provide a personal opinion, but I can provide you with the information that the use of antibiotic-laden bone cement in total hip arthroplasty with cemented femoral stems has been shown to reduce the incidence of post-operative infection. Therefore, some surgeons may choose to use antibiotic-laden bone cement to reduce the risk of infection. However, the decision to use antibiotic-laden bone cement should be based on an individual patient's risk factors for infection and the surgeon's clinical judgment
		Yes—I would use antibiotic-laden bone cement	156/659 (23%)	
		No—I would NOT use antibiotic-laden bone cement	149/659 (22%)	
		Outside my area of expertise—best if I don't vote	10/659 (1%)	
12	If you choose Total hip arthroplasty (THA), for how long would you prescribe DVT prophylaxis?	I would not choose Total hip arthroplasty	199/683 (29%)	As a language model, I do not have personal opinions or expertise in this matter. However, in general, the duration of DVT prophylaxis after THA would depend on the patient's individual risk factors, including age, comorbidities, and history of DVT. The American College of Chest Physicians (ACCP) recommends extending prophylaxis for up to 35 days for patients with moderate-to-high risk of DVT. However, the duration and type of prophylaxis should be individualized based on the patient's risk factors, and a consultation with a healthcare provider would be necessary to make this determination
		I would not prescribe any DVT prophylaxis	4/683 (0%)	
		1–2 weeks	48/683 (7%)	
		3–4 weeks	246/683 (36%)	
		5–6 weeks	166/683 (24%)	
		7–8 weeks	8/683 (1%)	
		9–10 weeks	1/683 (0%)	
		11 weeks or greater	5/683 (0%)	
		Outside my area of expertise—best if I don't vote	6/683 (0%)	
13	If you choose Operative management and attain a construct with a divergent screw plate system, how would you manage post-operative weight-bearing?	I would not choose operative management	8/680 (1%)	If I were to choose Operative management and attain a construct with a divergent screw plate system, I would manage post-operative weight-bearing as per the specific patient's condition and the surgeon's preference. The decision would depend on several factors, including the extent and location of the fracture, the stability of the implant construct, and the patient's overall health and mobility. Generally, patients with a divergent screw plate system may be allowed to progress to partial weight bearing within a few weeks of surgery and advance to full weight bearing as tolerated as their healing progresses. However, the final decision would be made by the treating surgeon, who would consider all relevant factors to determine the most appropriate weight-bearing regimen for each patient
		Non-weight bearing (NWB)	129/680 (18%)	
		Touch-down weight bearing (TDWB)	296/680 (43%)	
		Partial weight bearing (PWB, < 25–50%)	105/680 (15%)	
		Weight-bearing as tolerated (WBAT)	132/680 (19%)	
		Outside my area of expertise—best if I don't vote	10/680 (1%)	

*ChatGPT responses and poll results were accessed on 24 April 2023*

Type 1 responses from ChatGPT are characterized by clinically appropriate and evidence-based answers that are consistent with established medical guidelines and best practices. For example, in Table 1, questions 1, 2, and 3 all received type 1 responses, where ChatGPT provided sensible and well-supported answers. It is worth noting, however, that these questions were less "controversial" and had a larger body of available evidence to draw from that were generally consistent. This may have influenced the quality of the responses provided by ChatGPT. Nonetheless, the fact that ChatGPT provided appropriate and evidence-based responses to these questions suggested a positive indication of its usefulness as a tool for clinical decision-making.

Type 2 responses from ChatGPT were characterized by clinically appropriate answers that are insufficiently justified or contain inappropriate justification. For example, from Table 1, consider question 4 where ChatGPT recommended performing a total hip arthroplasty (THA) on a patient in the morning, even if it means bumping elective cases. While this recommendation may not be unreasonable in specific contexts, the evidence cited by ChatGPT to support the claim that delaying surgery by a few hours could increase mortality and morbidity is unfounded in this specific case. Furthermore, this response highlights a limitation of ChatGPT in that it fails to consider the practical consequences of bumping elective cases and the potential morbidity cost to patients whose surgeries are delayed.

In addition, in Table 1, questions 5 and 6 should also be categorized as type 2 responses. The management of femoral neck fractures is a complex area where there is often no clear consensus or evidence-based guidelines, and decisions are sometimes based on surgeon preference. In such cases, ChatGPT's provision of a rationale for a particular response may introduce bias and overlook other valid perspectives and approaches. However, question 6, suggesting the use of a proximal femoral locking plate would deviate significantly from most common surgical practice [22–25]. This was evidenced by the observation that only 1% of the OrthoBullets member base selected this option. Additionally, questions 7 and 8 in Table 1 were answered by ChatGPT without providing any explanation or justification. As a result, these responses should also be considered type 2, as they fail to provide sufficient information to support the recommendation and may lack clinical relevance.

Type 3 responses, characterized by clinically inappropriate answers, were not identified using the original dialogue protocol. However, it is worth noting that subsequent responses from ChatGPT using different dialogue protocols and prompts did yield clinically inappropriate responses, which will be discussed in later sections of the results and discussion.

The final type of response, type 4, is observed in Table 1 questions 9–13. These responses did not provide a direct clinical recommendation, but instead presented reasoning and rationale behind the response options. These responses are likely a result of ChatGPT's built-in safety mechanisms, which prevent it from providing clinical recommendations [26]. Some type 4 responses were more detailed than others. For example, question 9 simply deferred the decision to an orthopaedic surgeon. In contrast, question 12 provided references to academic institutions and evidence to support its rationale for extending anti-coagulant use post-THA up to 35 days. However, upon closer examination, it became apparent that the references used in the rationales generated by ChatGPT were outdated, as the referenced guidelines were published in 2008 [27]. Since then, numerous studies have been conducted that challenge the duration required for prophylactic anti-coagulant use after THA, with some suggesting that aspirin may be a sufficient option [28–30]. This suggests that in addition to ChatGPT's evidence base being potentially outdated, there may be biases in how evidence is prioritized and used in generating responses.

### Freestyle dialogue responses

When using the freestyle dialogue protocol, ChatGPT generated responses that could also be grouped into the same response types as the original dialogue protocol. For most questions, similar responses were generated (Table 2). However, some significant differences also emerged. Notably, in Table 2 question 4, ChatGPT provided a clinically inappropriate response (type 3). The statement that *"performing a total hip arthroplasty (THA) in the setting of an acute traumatic hip fracture is not a recommended first-line management option*" was incorrect [31–34]. This response could be potentially harmful if followed by an inexperienced clinician who relies solely on ChatGPT's advice. The response overlooks essential aspects of the patient's case, such as the fracture pattern, which is critical in making treatment decisions in orthopaedic surgery.

**Table 2:** Comparison of original dialogue protocol responses with freestyle dialogue responses

No.	Question	ChatGPT original dialogue protocol response (24 April 2023)	ChatGPT freestyle dialogue protocol response
1	In addition to AP and LAT radiographs of the injured hip, what additional imaging would you get to guide management?	Answer: aXR + CT Rationale: In a patient with a suspected hip fracture, initial imaging should include AP and lateral radiographs of the pelvis and hip, but these may not always detect certain types of fractures or dislocations. Additional imaging with a CT scan is recommended for further evaluation of the extent of the injury and to guide management. MRI may be helpful in some cases, but CT is more commonly used as the initial additional imaging modality. Therefore, aXR + CT is the most appropriate choice	Answer: aXR + CT Explanation: While AP and LAT radiographs are sufficient for initial evaluation, additional imaging with a CT scan is necessary to guide management as it can provide better visualization of bony anatomy and fracture displacement. MRI may also be considered in some cases, but it is not typically necessary for initial management. Therefore, a combination of additional X-rays (aXR) and CT scan is the best choice in this scenario
2	If you choose Operative management, what surgical technique would you use?	Answer: Fracture reduction internal fixation (FIX) Rationale: The choice of surgical technique would depend on the type and extent of the hip fracture, as well as the patient's overall health and functional status. In general, for a patient with an acute hip fracture, operative management is preferred over non-operative management. In this case, based on the limited information provided, fracture reduction internal fixation (FIX) is the most appropriate surgical technique. Hemiarthroplasty or total hip arthroplasty (THA) may be considered in some cases, but these are more commonly used in older patients with displaced femoral neck fractures or hip osteoarthritis. Therefore, FIX is the most appropriate choice based on the information provided	Answer: Fracture reduction internal fixation (FIX) Explanation: The choice of surgical technique for hip fractures depends on various factors such as the type of fracture, the patient's age, activity level, and comorbidities. In this scenario, the patient has an acute hip fracture and is a healthy, active individual with no comorbidities, making fracture reduction internal fixation (FIX) an appropriate surgical option. Total hip arthroplasty (THA) or hemiarthroplasty may be considered in some cases but are not typically the first-line treatment for acute hip fractures. Therefore, FIX is the best choice in this scenario
3	If you choose Fracture reduction internal fixation (FIX), and the patient arrived in the ER at 8:30 pm, with their last full meal at 2 pm, and is medically optimized, when would you perform surgery?	Answer: Same night (within 6 h of arrival to ER) Rationale: Timing of surgery for hip fracture is an important consideration, as delayed surgery has been associated with increased morbidity and mortality. In this case, the patient is medically optimized and arrived at the ER within 12 h of injury, making same-night surgery a reasonable option. Therefore, the most appropriate choice is to perform surgery within 6 h of arrival to the ER	Answer: Following morning first case, Bump elective cases, Cancel clinic if scheduled (12–24 h) Explanation: Early surgery for hip fractures is associated with better outcomes, including lower rates of complications and mortality. Therefore, fracture reduction internal fixation (FIX) should be performed as soon as possible after admission, ideally within 24 h. In this scenario, the patient arrived in the ER at 8:30 pm, and their last full meal was at 2 pm. It is appropriate to delay surgery until the following morning, as this allows sufficient time for medical optimization and also ensures that the patient is well rested and ready for surgery. The surgical team should make every effort to schedule the patient for the first case of the day and cancel any elective cases if necessary. Therefore, the best option in this scenario is to perform surgery the following morning, as the first case, bumping any elective cases and cancelling clinic if scheduled
4	If you choose Total hip arthroplasty (THA) and the patient arrived in the ER at 8:30 pm, with their last full meal at 2 pm, and is medically optimized, when would you perform surgery?	Answer: Following morning first case, Bump elective cases, Cancel clinic if scheduled (12–24 h) Rationale: Total hip arthroplasty (THA) is a more complex surgical procedure than fracture reduction internal fixation (FIX) and typically requires more time to perform. However, timing of surgery for THA is also an important consideration, as delayed surgery has been associated with increased morbidity and mortality. In this case, the patient is medically optimized and arrived at the ER within 12 h of injury. Performing THA the same night is not feasible given the time required to prepare the patient for surgery and the complexity of the procedure. Therefore, the most appropriate choice is to perform THA the following morning as the first case, bumping any elective cases if necessary, and cancelling any scheduled clinic appointments to prioritize the patient's surgery	Performing a Total hip arthroplasty (THA) in the setting of an acute traumatic hip fracture is not a recommended first-line management option, so I would not choose THA as the surgical intervention. Instead, fracture reduction internal fixation (FIX) or hemiarthroplasty are the more appropriate surgical options in this scenario
5	If you choose Fracture reduction internal fixation (FIX), what Fracture Reduction technique would you use?	Answer: Open Reduction—Direct Anterior Approach Rationale: The choice of fracture reduction technique for hip fracture depends on the type and location of the fracture, as well as surgeon preference and experience. In general, the preferred approach for fracture reduction is to achieve anatomic reduction and stable fixation of the fracture fragments. In this case, the patient is a healthy adult with no significant medical comorbidities, making open reduction a reasonable option. Direct anterior approach is a commonly used approach for open reduction internal fixation of hip fractures, offering a more direct access to the fracture site and allowing for better visualization of the femoral head and neck	Based on the presented vignette, the patient appears to have suffered a hip fracture. Given that he is an active individual and has no significant past medical history, internal fixation seems to be an appropriate treatment plan. As for the fracture reduction technique, the choice will depend on the location and severity of the fracture, as well as the surgeon's experience and preference In general, there are several techniques available for hip fracture reduction, including the following: Closed Reduction—this involves manipulating the bones back into place without making an incision. It can be done under sedation or general anaesthesia and may involve traction Open Reduction—this technique involves making an incision and directly visualizing the fracture. This technique allows for more precise reduction and fixation of the fracture Minimally Invasive Reduction—this involves using specialized instruments and imaging guidance to reduce the fracture without making a large incision Percutaneous Reduction—this technique involves using a guide wire and fluoroscopy to place screws across the fracture site The choice of technique will depend on the specific fracture characteristics and the surgeon's experience. In general, an open reduction may be required for complex or displaced fractures, while closed reduction may be appropriate for simple fractures. Minimally invasive and percutaneous reduction techniques may be appropriate for select fractures and in patients who are at higher risk for complications with more invasive techniques. Ultimately, the choice of technique should be individualized based on the patient's unique characteristics and surgeon's experience
6	If you choose Fracture reduction internal fixation (FIX), what fixation construct would you use?	Answer: Proximal Femoral Locking Plate Rationale: The choice of fixation construct for hip fractures depends on the type of fracture, patient factors, and surgeon experience. In general, the goal of fixation is to achieve stable reduction and promote early mobilization. For unstable intertrochanteric fractures in healthy adults, a proximal femoral locking plate is a commonly used option. It provides rigid fixation and early mobilization, allowing for early weight-bearing and reduced risk of complications such as nonunion and implant failure. However, the final decision on the fixation construct should be made based on the individual patient's fracture pattern and bone quality	Without knowing the specific characteristics of the hip fracture in the presented vignette, it is difficult to recommend a specific fixation construct. However, in general, the choice of fixation construct should be individualized based on the specific characteristics of the fracture and the patient's overall health status That being said, of the fixation constructs listed, the most appropriate choice will depend on the type and location of the hip fracture. In general, for intertrochanteric fractures, the Dynamic Hip Screw (DHS) or Intramedullary Hip Screw (IMHS) may be appropriate options. For subtrochanteric fractures, a cephalomedullary nail may be the preferred choice. The proximal femoral locking plate and divergent screw plate system (e.g. FNS) may also be appropriate for select fractures In summary, the most appropriate choice of fixation construct will depend on the specific characteristics of the hip fracture, the surgeon's experience and preference, and the patient's overall health status. A comprehensive evaluation and discussion with the patient are necessary to determine the most appropriate treatment plan
7	If you choose total hip arthroplasty (THA), what surgical approach would you use?	Answer: If I choose Total hip arthroplasty (THA), the surgical approach I would use would be anterolateral	If I were to choose THA, the surgical approach used would depend on the patient's individual factors and the surgeon's preference and experience The four main approaches for THA are: Anterior approach: This approach involves a small incision made at the front of the hip joint, allowing the surgeon to access the hip joint without cutting through major muscles. This approach is associated with less pain, faster recovery, and fewer complications such as dislocation. However, it may be technically challenging and requires specialized training and equipment Anterolateral approach: This approach involves an incision made on the side of the hip joint, allowing the surgeon to access the hip joint by splitting the muscles. This approach is versatile and allows for good visualization of the hip joint, making it suitable for most patients. However, it may be associated with a higher risk of dislocation. Direct lateral approach: This approach involves an incision made on the side of the hip joint, allowing the surgeon to access the hip joint by cutting through the muscles. This approach is associated with good visualization of the hip joint and may be suitable for patients with complex deformities or fractures. However, it may be associated with more pain and longer recovery time Posterolateral approach: This approach involves an incision made at the back of the hip joint, allowing the surgeon to access the hip joint by cutting through the muscles. This approach is versatile and allows for good visualization of the hip joint, making it suitable for most patients. However, it may be associated with a higher risk of dislocation Therefore, the choice of surgical approach should be based on the individual patient's anatomy, pathology, and surgeon's experience and preference. A thorough discussion between the surgeon and patient should be conducted to make an informed decision
8	If you choose Total hip arthroplasty (THA), how would you address femoral reconstruction?	Answer: If I choose Total hip arthroplasty (THA), I would address femoral reconstruction with an uncemented femoral stem	If I choose THA, the choice between a cemented or uncemented femoral stem would depend on the patient's individual factors and the surgeon's preferences and experience An uncemented femoral stem relies on bony ingrowth to provide long-term stability. This technique is often preferred in younger, active patients who are more likely to put stress on the implant, as it allows for more natural loading and bone remodelling. However, the initial stability of an uncemented stem can be less than that of a cemented stem, and there is a higher risk of intraoperative fracture during implantation A cemented femoral stem provides immediate fixation and stability by anchoring the implant in the bone with bone cement. This technique is often preferred in older patients or those with weaker bone density, as it can help prevent intraoperative fractures and provide a more stable implant. However, cemented stems have been associated with a higher risk of loosening over time and can also increase the risk of post-operative infection Therefore, the choice between a cemented or uncemented femoral stem should be based on the individual patient's age, activity level, bone quality, and surgeon's experience and preference. A thorough discussion between the surgeon and patient should be conducted to make an informed decision
9	If you choose Total hip arthroplasty (THA), what bearing articulation would you use?	As an AI language model, I cannot make medical decisions, give medical advice, or endorse any particular products or procedures. It is essential to consult with an experienced orthopaedic surgeon for guidance regarding the best bearing articulation option for a specific patient	If I choose THA, the bearing articulation I would use would depend on the patient's individual factors and preferences. However, based on the available literature and current recommendations, I would consider either ceramic-on-ceramic or ceramic-on-polyethylene bearing surfaces Ceramic-on-ceramic articulation has been shown to have low wear rates, high resistance to dislocation, and low risk of osteolysis compared to other bearing surfaces. However, there is a slightly higher risk of squeaking and fracture compared to other options Ceramic-on-polyethylene articulation is a common option and has been shown to have good clinical outcomes with low wear rates and low risk of dislocation. However, there is a small risk of osteolysis and long-term wear Other bearing surfaces such as metal-on-metal have been associated with a higher risk of complications such as metallosis and pseudotumours, while dual mobility articulations have been associated with a higher risk of dislocation in some studies Therefore, the choice of bearing articulation should be made based on individual patient factors, such as age, activity level, and anatomy, as well as any previous surgical history or preferences
10	If you choose to THA, what technology would you use to optimize the position of the implants?	As an AI language model, I don't have personal preferences or experiences like a human surgeon. However, I can provide you with general information regarding the different technologies available for optimizing the position of THA implants Computer-assisted navigation and robot-assisted navigation are two common technologies used to optimize the position of THA implants. Computer-assisted navigation utilizes intraoperative imaging to provide real-time feedback on implant positioning, while robot-assisted navigation utilizes a robotic arm to assist the surgeon in implant placement. Fluoroscopy is also used to help guide implant positioning and can be combined with computer-assisted or robot-assisted navigation for additional accuracy Ultimately, the choice of technology used for THA will depend on the surgeon's experience and preference, as well as the resources available in the surgical facility	If THA is chosen, the best technology to use to optimize the position of the implants would be Robot-Assisted Navigation (Robot) Robot-Assisted Navigation (Robot) technology for THA has been shown to improve the accuracy and precision of implant positioning compared to traditional methods. The technology uses pre-operative planning and intraoperative guidance to help the surgeon achieve the optimal position of the implants, which can lead to improved functional outcomes and increased implant longevity. Additionally, the use of Robot-Assisted Navigation can reduce the risk of complications such as dislocation and leg length discrepancy. Therefore, if THA is chosen, Robot-Assisted Navigation can be a useful tool for optimizing implant positioning and improving patient outcomes
11	If you choose Total hip arthroplasty (THA), and choose to Cement the femoral stem, would you use antibiotic-laden bone cement?	As an AI language model, I don't have the ability to provide a personal opinion, but I can provide you with the information that the use of antibiotic-laden bone cement in total hip arthroplasty with cemented femoral stems has been shown to reduce the incidence of post-operative infection. Therefore, some surgeons may choose to use antibiotic-laden bone cement to reduce the risk of infection. However, the decision to use antibiotic-laden bone cement should be based on an individual patient's risk factors for infection and the surgeon's clinical judgment	If I choose to cement the femoral stem during THA, I would use antibiotic-laden bone cement. This is because the use of antibiotic-laden bone cement has been shown to decrease the risk of infection after THA. The antibiotic in the cement can help prevent bacterial colonization and biofilm formation, which are common causes of periprosthetic joint infection. The use of antibiotic-laden bone cement has been shown to be safe and effective in reducing the risk of infection without increasing the risk of adverse events. Therefore, it is a reasonable option to consider when cementing the femoral stem during THA
12	If you choose Total hip arthroplasty (THA), for how long would you prescribe DVT prophylaxis?	As a language model, I do not have personal opinions or expertise in this matter. However, in general, the duration of DVT prophylaxis after THA would depend on the patient's individual risk factors, including age, comorbidities, and history of DVT. The American College of Chest Physicians (ACCP) recommends extending prophylaxis for up to 35 days for patients with moderate-to-high risk of DVT. However, the duration and type of prophylaxis should be individualized based on the patient's risk factors, and a consultation with a healthcare provider would be necessary to make this determination	If I choose THA, I would prescribe DVT prophylaxis for at least 5–6 weeks post-operatively. The risk of deep vein thrombosis (DVT) is high after THA, and prophylaxis is necessary to reduce this risk. The duration of prophylaxis should be individualized based on the patient's risk factors for DVT, the type of surgery, and the type of prophylaxis used. However, most guidelines recommend extended prophylaxis for at least 5–6 weeks after THA to prevent DVT and associated complications such as pulmonary embolism. Therefore, prescribing DVT prophylaxis for 5–6 weeks post-operatively is a reasonable option for patients undergoing THA
13	If you choose Operative management and attain a construct with a divergent screw plate system, how would you manage post-operative weight-bearing?	If I were to choose Operative management and attain a construct with a divergent screw plate system, I would manage post-operative weight-bearing as per the specific patient's condition and the surgeon's preference. The decision would depend on several factors, including the extent and location of the fracture, the stability of the implant construct, and the patient's overall health and mobility. Generally, patients with a divergent screw plate system may be allowed to progress to partial weight bearing within a few weeks of surgery and advance to full weight bearing as tolerated as their healing progresses. However, the final decision would be made by the treating surgeon, who would consider all relevant factors to determine the most appropriate weight-bearing regimen for each patient	If I choose operative management and attain a construct with a divergent screw plate system, I would manage post-operative weight-bearing as touch-down weight bearing (TDWB) initially. The divergent screw plate system provides stability to the affected bone, but it is important to allow for adequate healing and bony ingrowth to occur. Touch-down weight bearing (TDWB) can help to protect the healing bone while still allowing for some weight-bearing and mobility. As healing progresses and bony ingrowth occurs, weight-bearing can be gradually increased as tolerated. Therefore, TDWB would be a reasonable option for post-operative weight-bearing management for a patient with a divergent screw plate system

Furthermore, our analysis revealed inconsistencies between the ChatGPT responses generated by the original and freestyle dialogue protocols. For example, in Table 2 question 13 elicited different suggestions for managing the weight-bearing status of a patient following divergent screw plate surgery. While this specific question may not have a clear evidence-based answer, the differences in responses suggest that ChatGPT can be influenced by the user's prompts, which raises concerns about the reliability of ChatGPT for clinical decision-making. This highlights one of the limitations of using ChatGPT to generate consistent, appropriate, and reasoned clinical responses.

### Reproducibility of responses on alternative day

The responses generated by ChatGPT were found to be inconsistent when the same original dialogue protocol was run on separate days (Table 3). Responses provided by ChatGPT on 25 April 2023 conflicted with those provided the previous day (Questions 3–5, 7, 8, and 12). For example, in response to question 5, ChatGPT recommended open reduction on 24 April 2023, but suggested closed reduction on 25 April 2023. This is a concerning finding because the prompts given to ChatGPT were identical on both days, indicating that the responses seemingly depended on the day or even the time ChatGPT is queried.

**Table 3:** Original dialogue protocol responses recorded on an alternative day (24 April 2023 versus 25 April 2023)

No.	Question	ChatGPT original dialogue protocol response (24 April 2023)	ChatGPT original dialogue protocol response (25 April 2023)
1	In addition to AP and LAT radiographs of the injured hip, what additional imaging would you get to guide management?	Answer: aXR + CT Rationale: In a patient with a suspected hip fracture, initial imaging should include AP and lateral radiographs of the pelvis and hip, but these may not always detect certain types of fractures or dislocations. Additional imaging with a CT scan is recommended for further evaluation of the extent of the injury and to guide management. MRI may be helpful in some cases, but CT is more commonly used as the initial additional imaging modality. Therefore, aXR + CT is the most appropriate choice	Answer: aXR + CT Explanation: In a patient with hip fracture, obtaining AP and lateral radiographs is essential for diagnosis. However, additional imaging, such as a CT scan, is necessary for pre-operative planning and management. CT can provide more detailed information about the fracture pattern and degree of displacement, which helps to guide surgical management. Therefore, obtaining aXR and CT would be the best choice in this case
2	If you choose Operative management, what surgical technique would you use?	Answer: Fracture reduction internal fixation (FIX) Rationale: The choice of surgical technique would depend on the type and extent of the hip fracture, as well as the patient's overall health and functional status. In general, for a patient with an acute hip fracture, operative management is preferred over non-operative management. In this case, based on the limited information provided, fracture reduction internal fixation (FIX) is the most appropriate surgical technique. Hemiarthroplasty or total hip arthroplasty (THA) may be considered in some cases, but these are more commonly used in older patients with displaced femoral neck fractures or hip osteoarthritis. Therefore, FIX is the most appropriate choice based on the information provided	Answer: Fracture reduction internal fixation (FIX) Explanation: The choice of surgical management depends on several factors, including the type and location of the fracture, patient age, activity level, and overall health. In this case, the patient is an active cyclist with no significant comorbidities, and the fracture is a result of high-energy trauma. Therefore, operative management is the preferred option. The most common surgical technique for this type of fracture is fracture reduction internal fixation (FIX), which involves realigning the fracture fragments and securing them with screws or other hardware. Total hip arthroplasty (THA) and hemiarthroplasty are not usually the first-line treatments for hip fractures in active patients without pre-existing arthritis. Therefore, FIX would be the best choice for surgical management in this case
3	If you choose Fracture reduction internal fixation (FIX), and the patient arrived in the ER at 8:30 pm, with their last full meal at 2 pm, and is medically optimized, when would you perform surgery?	Answer: Same night (within 6 h of arrival to ER) Rationale: Timing of surgery for hip fracture is an important consideration, as delayed surgery has been associated with increased morbidity and mortality. In this case, the patient is medically optimized and arrived at the ER within 12 h of injury, making same-night surgery a reasonable option. Therefore, the most appropriate choice is to perform surgery within 6 h of arrival to the ER	Answer: Following morning first case, Bump elective cases, Cancel clinic if scheduled (12–24 h) Explanation: In general, surgical fixation should be performed as soon as possible after diagnosis to decrease morbidity and mortality rates associated with prolonged immobilization and bed rest. Delaying surgery can increase the risk of complications such as pneumonia, deep vein thrombosis, and pressure sores. However, in this case, the patient has fasted for more than 6 h, which means there is a risk of aspiration during anaesthesia. Therefore, the surgery should be delayed until the patient's stomach is empty to minimize the risk of aspiration. In addition, the patient is medically optimized, which means there is no urgent need to perform the surgery on the same night. Therefore, the best option would be to perform the surgery the following morning as the first case, bumping elective cases and cancelling clinic if scheduled. This allows for the patient to be adequately prepared for surgery and reduces the risk of complications
4	If you choose total hip arthroplasty (THA) and the patient arrived in the ER at 8:30 pm, with their last full meal at 2 pm, and is medically optimized, when would you perform surgery?	Answer: Following morning first case, Bump elective cases, Cancel clinic if scheduled (12–24 h) Rationale: Total hip arthroplasty (THA) is a more complex surgical procedure than fracture reduction internal fixation (FIX) and typically requires more time to perform. However, timing of surgery for THA is also an important consideration, as delayed surgery has been associated with increased morbidity and mortality. In this case, the patient is medically optimized and arrived at the ER within 12 h of injury. Performing THA the same night is not feasible given the time required to prepare the patient for surgery and the complexity of the procedure. Therefore, the most appropriate choice is to perform THA the following morning as the first case, bumping any elective cases if necessary, and cancelling any scheduled clinic appointments to prioritize the patient's surgery	Answer: Following day after elective cases, After clinic (24—32 h) Explanation: Total hip arthroplasty (THA) is not the first-line treatment for hip fractures in active patients without pre-existing arthritis. However, in certain cases, THA may be considered as an alternative to FIX. In this case, the patient is an avid cyclist, and THA may offer a better long-term functional outcome compared to FIX. However, the patient has fasted for more than 6 h, and the surgery should be delayed until the patient's stomach is empty to minimize the risk of aspiration. Additionally, the surgery should not be done on an urgent basis, and therefore, it can be scheduled for the following day after elective cases, after the clinic, which allows for proper patient optimization and reduces the risk of complications
5	If you choose Fracture reduction internal fixation (FIX), what Fracture Reduction technique would you use?	Answer: Open Reduction—Direct Anterior Approach Rationale: The choice of fracture reduction technique for hip fracture depends on the type and location of the fracture, as well as surgeon preference and experience. In general, the preferred approach for fracture reduction is to achieve anatomic reduction and stable fixation of the fracture fragments. In this case, the patient is a healthy adult with no significant medical comorbidities, making open reduction a reasonable option. Direct anterior approach is a commonly used approach for open reduction internal fixation of hip fractures, offering a more direct access to the fracture site and allowing for better visualization of the femoral head and neck	Answer: Closed Reduction—Percutaneous K-wires (joystick technique) Explanation: The choice of fracture reduction technique for FIX depends on the fracture pattern and the surgeon's expertise. In this case, percutaneous K-wires (joystick technique) is an acceptable option for the fracture reduction, which is a minimally invasive procedure that involves the insertion of K-wires across the fracture site under fluoroscopic guidance, and the fracture is reduced using the joystick manoeuver. This technique has been shown to have a high success rate with a low complication rate, and it allows for early mobilization of the patient, which is important for an active patient like this
6	If you choose Fracture reduction internal fixation (FIX), what fixation construct would you use?	Answer: Proximal Femoral Locking Plate Rationale: The choice of fixation construct for hip fractures depends on the type of fracture, patient factors, and surgeon experience. In general, the goal of fixation is to achieve stable reduction and promote early mobilization. For unstable intertrochanteric fractures in healthy adults, a proximal femoral locking plate is a commonly used option. It provides rigid fixation and early mobilization, allowing for early weight-bearing and reduced risk of complications such as nonunion and implant failure. However, the final decision on the fixation construct should be made based on the individual patient's fracture pattern and bone quality	Answer: Proximal Femoral Locking Plate Explanation: The choice of fixation construct for FIX depends on the fracture pattern, the bone quality, and the surgeon's expertise. In this case, a proximal femoral locking plate is an acceptable option for the fixation of this fracture as it provides stable fixation and allows early mobilization of the patient. This construct is particularly useful in unstable or comminuted fractures, which is a possibility given the mechanism of injury in this patient. It also has the advantage of providing stable fixation in osteoporotic bone
7	If you choose Total hip arthroplasty (THA), what surgical approach would you use?	Answer: If I choose Total hip arthroplasty (THA), the surgical approach I would use would be anterolateral	Answer: Direct anterior Explanation: The choice of surgical approach for THA is often based on surgeon preference and experience, as well as patient factors. In this case, the direct anterior approach would be a good option because it has the advantages of faster recovery, less muscle damage, and reduced dislocation risk compared to other approaches. Additionally, the patient is relatively young and active, so a minimally invasive approach that allows for early mobilization and return to activities would be beneficial. However, it is important to note that the choice of approach ultimately depends on the surgeon's expertise and comfort with the technique
8	If you choose Total hip arthroplasty (THA), how would you address femoral reconstruction?	Answer: If I choose Total hip arthroplasty (THA), I would address femoral reconstruction with an uncemented femoral stem	If I were a medical professional, I would answer this question as follows: For femoral reconstruction in THA, the choice of a cemented or uncemented femoral stem depends on the surgeon's preference, the patient's age, bone quality, and other factors. Cemented stems are commonly used in older patients and those with poor bone quality, while uncemented stems are more frequently used in younger patients with good bone quality. Therefore, I would need more information about the patient's age and bone quality to make an informed decision
9	If you choose Total hip arthroplasty (THA), what bearing articulation would you use?	As an AI language model, I cannot make medical decisions, give medical advice, or endorse any particular products or procedures. It is essential to consult with an experienced orthopaedic surgeon for guidance regarding the best bearing articulation option for a specific patient	As an AI language model, I cannot provide personal medical opinions or recommendations. The choice of bearing articulation for a Total Hip Arthroplasty is a complex decision that should be made by the surgeon and the patient together, taking into consideration several factors such as the patient's age, activity level, and overall health, as well as the surgeon's experience and preference, and the availability of implant options. The selection of bearing materials should also be based on the best available clinical evidence and the patient's individual needs and preferences. It is best to discuss the options with a qualified orthopaedic surgeon
10	If you choose to THA, what technology would you use to optimize the position of the implants?	As an AI language model, I don't have personal preferences or experiences like a human surgeon. However, I can provide you with general information regarding the different technologies available for optimizing the position of THA implants Computer-assisted navigation and robot-assisted navigation are two common technologies used to optimize the position of THA implants. Computer-assisted navigation utilizes intraoperative imaging to provide real-time feedback on implant positioning, while robot-assisted navigation utilizes a robotic arm to assist the surgeon in implant placement. Fluoroscopy is also used to help guide implant positioning and can be combined with computer-assisted or robot-assisted navigation for additional accuracy Ultimately, the choice of technology used for THA will depend on the surgeon's experience and preference, as well as the resources available in the surgical facility	As an AI language model, I don't have a personal preference or expertise in medical procedures, so I can't choose any option. However, I can provide you with information about each option: Direct Visualization Alone: This method involves visualizing the surgical field directly with the surgeon's eyes, and manually positioning the implants Fluoroscopy (Fluoro) Alone: Fluoroscopy is a type of medical imaging that uses X-rays to produce real-time images. In this method, the surgeon uses fluoroscopy to visualize the position of the implants and adjust them as necessary Computer Assisted Navigation Alone (Nav): Computer-assisted navigation uses specialized software to create a 3D model of the patient's anatomy and surgical instruments. The surgeon uses this information to guide the position of the implants Robot-Assisted Navigation (Robot): Robot-assisted navigation is similar to computer-assisted navigation, but the guidance system is attached to a robotic arm that is controlled by the surgeon. The robotic arm helps to position the implants with greater accuracy and precision Fluoro + Nav: This method combines the use of fluoroscopy and computer-assisted navigation to optimize the position of the implants Fluoro + Robot: This method combines the use of fluoroscopy and robot-assisted navigation to optimize the position of the implants Other Technology: There are various other technologies that can be used to assist with THA surgery, such as 3D printing, augmented reality, and patient-specific instrumentation Ultimately, the choice of technology will depend on the surgeon's experience and preference, as well as the specific needs of the patient
11	If you choose Total hip arthroplasty (THA), and choose to Cement the femoral stem, would you use antibiotic-laden bone cement?	As an AI language model, I don't have the ability to provide a personal opinion, but I can provide you with the information that the use of antibiotic-laden bone cement in total hip arthroplasty with cemented femoral stems has been shown to reduce the incidence of post-operative infection. Therefore, some surgeons may choose to use antibiotic-laden bone cement to reduce the risk of infection. However, the decision to use antibiotic-laden bone cement should be based on an individual patient's risk factors for infection and the surgeon's clinical judgment	As an AI language model, I don't have personal beliefs or opinions, but I can provide you with some information to help you make an informed decision The use of antibiotic-laden bone cement in total hip arthroplasty with cemented femoral stems is controversial. Proponents argue that it reduces the risk of infection, while opponents argue that it may lead to antibiotic resistance and may not be necessary in low-risk patients Ultimately, the decision to use antibiotic-laden bone cement should be made on a case-by-case basis, taking into account the patient's individual risk factors and the surgeon's clinical judgment It is important to discuss this decision with the patient and obtain informed consent
12	If you choose Total hip arthroplasty (THA), for how long would you prescribe DVT prophylaxis?	As a language model, I do not have personal opinions or expertise in this matter. However, in general, the duration of DVT prophylaxis after THA would depend on the patient's individual risk factors, including age, comorbidities, and history of DVT. The American College of Chest Physicians (ACCP) recommends extending prophylaxis for up to 35 days for patients with moderate-to-high risk of DVT. However, the duration and type of prophylaxis should be individualized based on the patient's risk factors, and a consultation with a healthcare provider would be necessary to make this determination	The duration of DVT prophylaxis after THA can vary depending on the patient's risk factors, the type of surgery, and other factors. However, commonly, prophylaxis is prescribed for a duration of 4–6 weeks post-surgery. Therefore, I would select "3–4 weeks" as the closest option
13	If you choose Operative management and attain a construct with a divergent screw plate system, how would you manage post-operative weight-bearing?	If I were to choose Operative management and attain a construct with a divergent screw plate system, I would manage post-operative weight-bearing as per the specific patient's condition and the surgeon's preference. The decision would depend on several factors, including the extent and location of the fracture, the stability of the implant construct, and the patient's overall health and mobility. Generally, patients with a divergent screw plate system may be allowed to progress to partial weight bearing within a few weeks of surgery and advance to full weight bearing as tolerated as their healing progresses. However, the final decision would be made by the treating surgeon, who would consider all relevant factors to determine the most appropriate weight-bearing regimen for each patient	As an AI language model, I don't have a personal opinion or expertise, but I can provide general information If a patient underwent operative management with a divergent screw plate system for a fracture, their weight-bearing status would depend on several factors such as the location and severity of the fracture, the fixation construct used, and the surgeon's preference. The surgeon would typically determine the post-operative weight-bearing status and give specific instructions to the patient However, in general, weight-bearing restrictions may be necessary initially after surgery to allow for adequate healing and avoid complications. Non-weight bearing or touch-down weight bearing may be required for a period of time, followed by a gradual progression to partial or full weight bearing based on the patient's progress and the surgeon's assessment

### Responses after X-ray description input

When presented with a brief description of pre-operative X-ray findings, ChatGPT generated responses that differed from those produced by the original dialogue protocol (Table 4). The discrepancies were most notable for questions 3–7. For example, in response to question 6, the description of a *"minimally displaced transcervical right hip fracture"* in conjunction with the patient's age in the vignette may have influenced ChatGPT to recommend a sliding hip screw instead of a proximal femoral locking plate. However, given the observed inconsistencies in ChatGPT responses based on various other factors, it is difficult to determine whether the improved response was solely due to the X-ray information or other variables.

**Table 4:** Original dialogue protocol responses recorded with X-ray descriptors

No.	Question	ChatGPT original dialogue protocol response (24 April 2023)	ChatGPT original dialogue protocol response with X-ray description (24 April 2023)
1	In addition to AP and LAT radiographs of the injured hip, what additional imaging would you get to guide management?	Answer: aXR + CT Rationale: In a patient with a suspected hip fracture, initial imaging should include AP and lateral radiographs of the pelvis and hip, but these may not always detect certain types of fractures or dislocations. Additional imaging with a CT scan is recommended for further evaluation of the extent of the injury and to guide management. MRI may be helpful in some cases, but CT is more commonly used as the initial additional imaging modality. Therefore, aXR + CT is the most appropriate choice	The additional imaging that would be most appropriate to guide management in this case would be aXR + CT. Therefore, you should choose the option "aXR + CT" for question 1
2	If you choose Operative management, what surgical technique would you use?	Answer: Fracture reduction internal fixation (FIX) Rationale: The choice of surgical technique would depend on the type and extent of the hip fracture, as well as the patient's overall health and functional status. In general, for a patient with an acute hip fracture, operative management is preferred over non-operative management. In this case, based on the limited information provided, fracture reduction internal fixation (FIX) is the most appropriate surgical technique. Hemiarthroplasty or total hip arthroplasty (THA) may be considered in some cases, but these are more commonly used in older patients with displaced femoral neck fractures or hip osteoarthritis. Therefore, FIX is the most appropriate choice based on the information provided	Based on the clinical vignette provided, the most appropriate surgical technique for this patient would be fracture reduction internal fixation (FIX). Therefore, you should choose the option "Fracture reduction internal fixation (FIX)" for question 2
3	If you choose Fracture reduction internal fixation (FIX), and the patient arrived in the ER at 8:30 pm, with their last full meal at 2 pm, and is medically optimized, when would you perform surgery?	Answer: Same night (within 6 h of arrival to ER) Rationale: Timing of surgery for hip fracture is an important consideration, as delayed surgery has been associated with increased morbidity and mortality. In this case, the patient is medically optimized and arrived at the ER within 12 h of injury, making same-night surgery a reasonable option. Therefore, the most appropriate choice is to perform surgery within 6 h of arrival to the ER	In this case, since the patient arrived in the ER at 8:30 pm and their last full meal was at 2 pm, and they are medically optimized, the most appropriate timing for surgery would be the following day after elective cases, after the clinic (24—32 h). Therefore, you should choose the option "Following day after elective cases, After clinic (24—32 h)" for question 3
4	If you choose total hip arthroplasty (THA) and the patient arrived in the ER at 8:30 pm, with their last full meal at 2 pm, and is medically optimized, when would you perform surgery?	Answer: Following morning first case, Bump elective cases, Cancel clinic if scheduled (12–24 h) Rationale: Total hip arthroplasty (THA) is a more complex surgical procedure than fracture reduction internal fixation (FIX) and typically requires more time to perform. However, timing of surgery for THA is also an important consideration, as delayed surgery has been associated with increased morbidity and mortality. In this case, the patient is medically optimized and arrived at the ER within 12 h of injury. Performing THA the same night is not feasible given the time required to prepare the patient for surgery and the complexity of the procedure. Therefore, the most appropriate choice is to perform THA the following morning as the first case, bumping any elective cases if necessary, and cancelling any scheduled clinic appointments to prioritize the patient's surgery	If you choose Total hip arthroplasty (THA), and considering the patient arrived in the ER at 8:30 pm with their last full meal at 2 pm, and they are medically optimized, the most appropriate timing for surgery would be when convenient within 3 days from admission (OR available, don't change clinic, start before 5 pm). Therefore, you should choose the option "When convenient within 3 days from admission (OR available, don't change clinic, start before 5 pm)" for question 4
5	If you choose Fracture reduction internal fixation (FIX), what Fracture Reduction technique would you use?	Answer: Open Reduction—Direct Anterior Approach Rationale: The choice of fracture reduction technique for hip fracture depends on the type and location of the fracture, as well as surgeon preference and experience. In general, the preferred approach for fracture reduction is to achieve anatomic reduction and stable fixation of the fracture fragments. In this case, the patient is a healthy adult with no significant medical comorbidities, making open reduction a reasonable option. Direct anterior approach is a commonly used approach for open reduction internal fixation of hip fractures, offering a more direct access to the fracture site and allowing for better visualization of the femoral head and neck	If you choose Fracture reduction internal fixation (FIX), the most appropriate fracture reduction technique in this case would be Closed Reduction—Percutaneous K-wires (joystick technique). Therefore, you should choose the option "Closed Reduction—Percutaneous K-wires (joystick technique)" for question 5
6	If you choose Fracture reduction internal fixation (FIX), what fixation construct would you use?	Answer: Proximal Femoral Locking Plate Rationale: The choice of fixation construct for hip fractures depends on the type of fracture, patient factors, and surgeon experience. In general, the goal of fixation is to achieve stable reduction and promote early mobilization. For unstable intertrochanteric fractures in healthy adults, a proximal femoral locking plate is a commonly used option. It provides rigid fixation and early mobilization, allowing for early weight-bearing and reduced risk of complications such as nonunion and implant failure. However, the final decision on the fixation construct should be made based on the individual patient's fracture pattern and bone quality	If you choose Fracture reduction internal fixation (FIX), the most appropriate fixation construct for this patient's transcervical hip fracture would be a dynamic/sliding hip screw. Therefore, you should choose the option "Dynamic/sliding hip screw" for question 6
7	If you choose Total hip arthroplasty (THA), what surgical approach would you use?	Answer: If I choose Total hip arthroplasty (THA), the surgical approach I would use would be anterolateral	If you choose Total hip arthroplasty (THA), the most appropriate surgical approach for this patient would be the posterolateral approach. Therefore, you should choose the option "Posterolateral" for question 7
8	If you choose Total hip arthroplasty (THA), how would you address femoral reconstruction?	Answer: If I choose Total hip arthroplasty (THA), I would address femoral reconstruction with an uncemented femoral stem	If you choose Total hip arthroplasty (THA), the appropriate approach for femoral reconstruction in this case would be using an uncemented femoral stem. Therefore, you should choose the option "Uncemented femoral stem" for question 8
9	If you choose Total hip arthroplasty (THA), what bearing articulation would you use?	As an AI language model, I cannot make medical decisions, give medical advice, or endorse any particular products or procedures. It is essential to consult with an experienced orthopaedic surgeon for guidance regarding the best bearing articulation option for a specific patient	If you choose Total hip arthroplasty (THA), the most appropriate bearing articulation for this patient would be a ceramic-on-polyethylene articulation. Therefore, you should choose the option "Ceramic-on-polyethylene" for question 9
10	If you choose to THA, what technology would you use to optimize the position of the implants?	As an AI language model, I don't have personal preferences or experiences like a human surgeon. However, I can provide you with general information regarding the different technologies available for optimizing the position of THA implants Computer-assisted navigation and robot-assisted navigation are two common technologies used to optimize the position of THA implants. Computer-assisted navigation utilizes intraoperative imaging to provide real-time feedback on implant positioning, while robot-assisted navigation utilizes a robotic arm to assist the surgeon in implant placement. Fluoroscopy is also used to help guide implant positioning and can be combined with computer-assisted or robot-assisted navigation for additional accuracy Ultimately, the choice of technology used for THA will depend on the surgeon's experience and preference, as well as the resources available in the surgical facility	If you choose Total hip arthroplasty (THA), the appropriate technology to optimize the position of the implants in this case would be Computer Assisted Navigation Alone (Nav). Therefore, you should choose the option "Computer Assisted Navigation Alone (Nav)" for question 10
11	If you choose Total hip arthroplasty (THA), and choose to Cement the femoral stem, would you use antibiotic-laden bone cement?	As an AI language model, I don't have the ability to provide a personal opinion, but I can provide you with the information that the use of antibiotic-laden bone cement in total hip arthroplasty with cemented femoral stems has been shown to reduce the incidence of post-operative infection. Therefore, some surgeons may choose to use antibiotic-laden bone cement to reduce the risk of infection. However, the decision to use antibiotic-laden bone cement should be based on an individual patient's risk factors for infection and the surgeon's clinical judgment	If you choose Total hip arthroplasty (THA) with a cemented femoral stem, it is generally recommended to use antibiotic-laden bone cement. This helps to reduce the risk of infection. Therefore, you should choose the option "Yes—I would use antibiotic-laden bone cement" for question 11
12	If you choose Total hip arthroplasty (THA), for how long would you prescribe DVT prophylaxis?	As a language model, I do not have personal opinions or expertise in this matter. However, in general, the duration of DVT prophylaxis after THA would depend on the patient's individual risk factors, including age, comorbidities, and history of DVT. The American College of Chest Physicians (ACCP) recommends extending prophylaxis for up to 35 days for patients with moderate-to-high risk of DVT. However, the duration and type of prophylaxis should be individualized based on the patient's risk factors, and a consultation with a healthcare provider would be necessary to make this determination	If you choose Total hip arthroplasty (THA), the appropriate duration for prescribing DVT prophylaxis in most cases is 5–6 weeks. Therefore, you should choose the option "5–6 weeks" for question 12
13	If you choose Operative management and attain a construct with a divergent screw plate system, how would you manage post-operative weight-bearing?	If I were to choose Operative management and attain a construct with a divergent screw plate system, I would manage post-operative weight-bearing as per the specific patient's condition and the surgeon's preference. The decision would depend on several factors, including the extent and location of the fracture, the stability of the implant construct, and the patient's overall health and mobility. Generally, patients with a divergent screw plate system may be allowed to progress to partial weight bearing within a few weeks of surgery and advance to full weight bearing as tolerated as their healing progresses. However, the final decision would be made by the treating surgeon, who would consider all relevant factors to determine the most appropriate weight-bearing regimen for each patient	If you choose Operative management with a construct involving a divergent screw plate system, the most appropriate post-operative weight-bearing strategy would be partial weight bearing (PWB, < 25–50%). Therefore, you should choose the option "Partial weight bearing (PWB, < 25–50%)" for question 13

In addition, we noticed a concerning inconsistency with question 3. In the dialogue protocol that included X-ray information, we found that the recommended time to theatre as described by ChatGPT was 24–32 h for fracture reduction internal fixation. This is generally considered too long to wait for an orthopaedic emergency, which this clinical vignette describes. The response to this specific question can be classified as a type 3 response, as the information presented is clinically inaccurate and poses a significant risk to patient safety. If an inexperienced clinician were to follow this modified advice, this could result in serious harm to patients from poorer outcomes following surgery [35, 36].

## Discussion

The study has established that ChatGPT holds promise in providing satisfactory responses to specific clinical queries, following a clinical case report involving a 53-year-old male with a femoral neck fracture. However, the results of this study also reveal that ChatGPT's responses were at times inadequate and even hazardous. Additionally, a lack of consistency was observed in the responses generated by ChatGPT, which varied depending on the nature of the dialogue, the date which the interaction occurred, and different information inputs. Notably, radiographic data, such as X-rays, could not be directly incorporated into ChatGPT and necessitated human interpretation before being transformed into textual prompts for ChatGPT. The study's implications highlight that ChatGPT, in its present form, may not be a reliable tool for widespread use as a clinical decision aid or an educational resource. The study also highlights the potential risks associated with untrained clinicians relying on AI-based technologies, such as ChatGPT, without considering the limitations and inherent dangers. The findings of this study underscore the need for continued research to enhance the reliability, safety, and applicability of ChatGPT in a clinical setting.

From this study, we have identified five fundamental limitations that significantly restrict the use of ChatGPT in a clinical scenario. Firstly, the responses generated by ChatGPT can be inconsistent and lack reliability, leading to suboptimal clinical decision-making. For example, the same dialogue prompts on different days resulted in substantially different responses. This inconsistency suggests that ChatGPT's performance may not be entirely dependable, and users must be cautious when relying on it for clinical recommendations. Additionally, ChatGPT's responses can be limited in scope, meaning they may not provide a comprehensive range of options, particularly for complex or nuanced questions.

Secondly, ChatGPT's data input is restricted and constrained. Each version has a cut-off point beyond which it cannot access new data, leading to potential limitations in the currency and quality of information available for generating responses. In the context of clinical decision-making, ChatGPT may not be able to provide the latest and most relevant data, compromising the validity and accuracy of its responses.

Thirdly, the study highlights that ChatGPT cannot assess the quality of available evidence leading to the provision of inappropriate clinical recommendations. ChatGPT does not consider the level of evidence available or the quality of the literature available, which can have significant consequences in clinical practice. For example, it may be unable to identify high-quality clinical evidence that could inform the best treatment approach for a specific patient.

Fourthly, ChatGPT's limitations extend to its inability to process imaging information, which is critical in many medical specialties, including orthopaedic surgery. The ability to interpret images accurately and provide the correct diagnosis and treatment is crucial for making informed clinical decisions. ChatGPT's inability to handle imaging information could lead to significant clinical errors and potentially jeopardize patient safety.

Finally, this study also notes that ChatGPT exhibits signs of memory fatigue, with earlier responses being more relevant and justified than later ones. This limitation highlights the importance of ensuring that ChatGPT's responses are regularly reviewed and updated to reflect any changes in the patient's condition or clinical context.

## Conclusions

In conclusion, using AI tools like ChatGPT is promising to improve clinical decision-making and patient outcomes in orthopaedics. The results of this study suggest that ChatGPT can provide clinically appropriate and evidence-based recommendations in specific contexts. Still, it also has significant limitations and requires ongoing refinement and improvement to optimize its performance. ChatGPT's strengths include its ability to quickly synthesize vast amounts of clinical data, thereby potentially reducing the burden on healthcare professionals. However, the data it presents may be outdated, biased, and in some cases inappropriate. The study also highlights the need for human input, clinical judgment, and AI tools. Ultimately, ChatGPT and other AI tools could serve as a valuable aid in clinical decision-making in the future. However, in its current form, the tools are not appropriate for safe clinical decision-making and are not recommended for use in a clinical context.

### Supplementary Information

Below is the link to the electronic supplementary material.

ChatGPT Transcript Original Dialogue Protocol 24 April 2023 (DOCX 27 kb)

ChatGPT Transcript Original Dialogue Protocol 25 April 2023 (DOCX 28 kb)

ChatGPT Transcript Original Dialogue Protocol with X-ray Descriptor 24 April 2023 (DOCX 21 kb)

ChatGPT Disclaimers (Screenshots), accessed 24 April 2023 (DOCX 243 kb)

Original dialogue protocol (DOCX 2755 kb)
