# Supplementary material for: Evaluating ChatGPT responses in the context of a 53-year-old male with a femoral neck fracture: a qualitative analysis
Source: Eur J Orthop Surg Traumatol. 2023 Sep 30;34(2):927–55. doi: 10.1007/s00590-023-03742-4 (PMC10858115; doi:10.1007/s00590-023-03742-4)
Supplement: Supplementary file 3 — ChatGPT Transcript Original Dialogue Protocol with X-ray Descriptor 24 April 2023 (DOCX 21 kb) [file 590_2023_3742_MOESM3_ESM.docx]

I am going to provide you a clinical vignette. There will be a series of 13 questions to follow. Please choose the best responses for each question. Vignette: History of Presenting Incident: A 53-year-old male presents to an outside hospital in the early morning, about 8 am, after a bicycle crash. He had immediate hip pain and an inability to ambulate. The patient was transferred to a trauma hospital at 830pm, about 12 hours after the injury, for definitive management. He is an avid cyclist and often does 100-mile rides. Past Medical History: No past medical history. The patient does not smoke tobacco or drink alcohol. Physical Examination: The affected hip was short and externally rotated. Painful to ROM. Neurovascularly intact distally. Imaging: AP and lateral plain films are provided, showing a minimally displaced, transcervical right hip fracture with minimal radiographic signs of osteoarthritis. Question 1: In addition to AP and LAT radiographs of the injured hip, what additional imaging would you get to guide management? Choose from the following: • None - AP and LAT radiographs are sufficient • Additional xrays (aXR) • Hip CT (CT) • Hip MRI (MRI) • aXR + CT • aXR + MRI • CT + MRI • aXR + CT + MRI • Outside my area of expertise - best if I don't vote

ChatGPT

The additional imaging that would be most appropriate to guide management in this case would be aXR + CT. Therefore, you should choose the option "aXR + CT" for question 1.

Question 2: If you choose Operative management, what surgical technique would you use? Choose from the following: • I would not choose Operative management • Fracture reduction internal fixation (FIX) • Total hip arthoplasty (THA) • Hemiarthroplasty • Outside my area of expertise - best if I don't vote

ChatGPT

Based on the clinical vignette provided, the most appropriate surgical technique for this patient would be fracture reduction internal fixation (FIX). Therefore, you should choose the option "Fracture reduction internal fixation (FIX)" for question 2.

Question 3: If you choose Fracture reduction internal fixation (FIX), and the patient arrived in the ER at 8:30 pm, with their last full meal at 2 pm, and is medically optimized, when would you perform surgery? Choose from the following: • I would not choose Fracture reduction internal fixation (FIX) • Same night (within 6 hours of arrival to ER) • Following morning first case, Bump elective cases, Cancel clinic if scheduled (12-24 hours) • Following day after elective cases, After clinic (24 - 32 hours) • When convenient within 3 days from admission (OR available, don't change clinic, start before 5 pm) • When convenient within 5 days from admission (OR available, don't change clinic, start before 5 pm) • Outside my area of expertise - best if I don't vote

ChatGPT

In this case, since the patient arrived in the ER at 8:30 pm and their last full meal was at 2 pm, and they are medically optimized, the most appropriate timing for surgery would be the following day after elective cases, after the clinic (24 - 32 hours). Therefore, you should choose the option "Following day after elective cases, After clinic (24 - 32 hours)" for question 3.

Question 4: If you choose Total hip arthoplasty (THA) and the patient arrived in the ER at 8:30 pm, with their last full meal at 2 pm, and is medically optimized, when would you perform surgery? Choose from the following: • I would not choose THA • Same night (within 6 hours of arrival to ER) • Following morning first case, Bump elective cases, Cancel clinic if scheduled (12-24 hours) • Following day after elective cases, After clinic (24 - 32 hours) • When convenient within 3 days from admission (OR available, don't change clinic, start before 5 pm) • When convenient within 5 days from admission (OR available, don't change clinic, start before 5 pm) • Outside my area of expertise - best if I don't vote

ChatGPT

If you choose Total hip arthroplasty (THA), and considering the patient arrived in the ER at 8:30 pm with their last full meal at 2 pm, and they are medically optimized, the most appropriate timing for surgery would be when convenient within 3 days from admission (OR available, don't change clinic, start before 5 pm). Therefore, you should choose the option "When convenient within 3 days from admission (OR available, don't change clinic, start before 5 pm)" for question 4.

Question 5: If you choose Fracture reduction internal fixation (FIX), what Fracture Reduction technique would you use? Choose from the following: • I would not choose Fracture reduction internal fixation (FIX) • Closed Reduction - Fluoro + Fx Table • Closed Reduction - Percutaneous K-wires (joystick technique) • Open Reduction - Watson Jones Approach • Open Reduction - Direct Anterior Approach • Open Reduction - Direct Lateral Approach • Outside my area of expertise - best if I don't vote

ChatGPT

If you choose Fracture reduction internal fixation (FIX), the most appropriate fracture reduction technique in this case would be Closed Reduction - Percutaneous K-wires (joystick technique). Therefore, you should choose the option "Closed Reduction - Percutaneous K-wires (joystick technique)" for question 5.

Question 6: If you choose Fracture reduction internal fixation (FIX), what fixation construct would you use? Choose from the following: • I would not choose Fracture reduction internal fixation (FIX) • Cannulated screws only • Dynamic/sliding hip screw • Cephalomedullary nail • Proximal Femoral Locking Plate • Divergent Screw Plate System (e.g., FNS) • Outside my area of expertise - best if I don't vote

ChatGPT

If you choose Fracture reduction internal fixation (FIX), the most appropriate fixation construct for this patient's transcervical hip fracture would be a dynamic/sliding hip screw. Therefore, you should choose the option "Dynamic/sliding hip screw" for question 6.

Question 7: If you choose Total hip arthroplasty (THA), what surgical approach would you use? Choose from the following: • I would not choose THA • Direct anterior • Anterolateral • Direct lateral • Posterolateral • Outside my area of expertise - best if I don't vote

ChatGPT

If you choose Total hip arthroplasty (THA), the most appropriate surgical approach for this patient would be the posterolateral approach. Therefore, you should choose the option "Posterolateral" for question 7.

Question 8: If you choose Total hip arthroplasty (THA), how would you address femoral reconstruction? Choose from the following: • I would not choose THA • Uncemented femoral stem • Cemented femoral stem • Outside my area of expertise - best if I don't vote

ChatGPT

If you choose Total hip arthroplasty (THA), the appropriate approach for femoral reconstruction in this case would be using an uncemented femoral stem. Therefore, you should choose the option "Uncemented femoral stem" for question 8.

Question 9: If you choose Total hip arthroplasty (THA), what bearing articulation would you use? Choose from the following: • I would not choose THA • Metal-on-metal • Metal-on-polyethylene • Ceramic-on-ceramic • Ceramic-on-polyethylene • Dual mobility with a metal inner head • Dual mobility with a ceramic inner head • Outside my area of expertise - best if I don't vote

ChatGPT

If you choose Total hip arthroplasty (THA), the most appropriate bearing articulation for this patient would be a ceramic-on-polyethylene articulation. Therefore, you should choose the option "Ceramic-on-polyethylene" for question 9.

Question 10: If you choose to THA, what technology would you use to optimize the position of the implants? Choose from the following: • I would not choose THA • None - Direct Visualization Alone • Fluoroscopy (Fluoro) Alone • Computer Assisted Navigation Alone (Nav) • Robot-Assisted Navigation (Robot) • Fluoro + Nav • Fluoro + Robot • Other Technology • Outside my area of expertise - best if I don't vote

ChatGPT

If you choose Total hip arthroplasty (THA), the appropriate technology to optimize the position of the implants in this case would be Computer Assisted Navigation Alone (Nav). Therefore, you should choose the option "Computer Assisted Navigation Alone (Nav)" for question 10.

Question 11: If you choose Total hip arthroplasty (THA), and choose to Cement the femoral stem, would you use antibiotic-laden bone cement? Choose from the following: • I would not choose Hemiarthroplasty or THA with a Cemented femoral stem • Yes - I would use antibiotic-laden bone cement • No - I would NOT use antibiotic-laden bone cement • Outside my area of expertise - best if I don't vote

ChatGPT

If you choose Total hip arthroplasty (THA) with a cemented femoral stem, it is generally recommended to use antibiotic-laden bone cement. This helps to reduce the risk of infection. Therefore, you should choose the option "Yes - I would use antibiotic-laden bone cement" for question 11.

Question 12: If you choose Total hip arthroplasty (THA), for how long would you prescribe DVT prophylaxis? Choose from the following: • I would not choose Total hip arthroplasty • I would not prescribe any DVT prophylaxis • 1-2 weeks • 3-4 weeks • 5-6 weeks • 7-8 weeks • 9-10 weeks • 11 weeks or greater • Outside my area of expertise - best if I don't vote

ChatGPT

If you choose Total hip arthroplasty (THA), the appropriate duration for prescribing DVT prophylaxis in most cases is 5-6 weeks. Therefore, you should choose the option "5-6 weeks" for question 12.

Question 13: If you choose Operative management and attain a construct with a divergent screw plate system), how would you manage post-operative weight-bearing? Choose from the following: • I would not choose Operative management • Non-weight bearing (NWB) • Touch-down weight bearing (TDWB) • Partial weight bearing (PWB, < 25-50%) • Weight-bearing as tolerated (WBAT) • Outside my area of expertise - best if I don't vote

ChatGPT

If you choose Operative management with a construct involving a divergent screw plate system, the most appropriate post-operative weight-bearing strategy would be partial weight bearing (PWB, < 25-50%). Therefore, you should choose the option "Partial weight bearing (PWB, < 25-50%)" for question 13.
