# Supplementary figures and images for: Evaluating ChatGPT responses in the context of a 53-year-old male with a femoral neck fracture: a qualitative analysis
Source: Eur J Orthop Surg Traumatol. 2023 Sep 30;34(2):927–55. doi: 10.1007/s00590-023-03742-4 (PMC10858115; doi:10.1007/s00590-023-03742-4)

Appendix 2 – ChatGPT Disclaimers


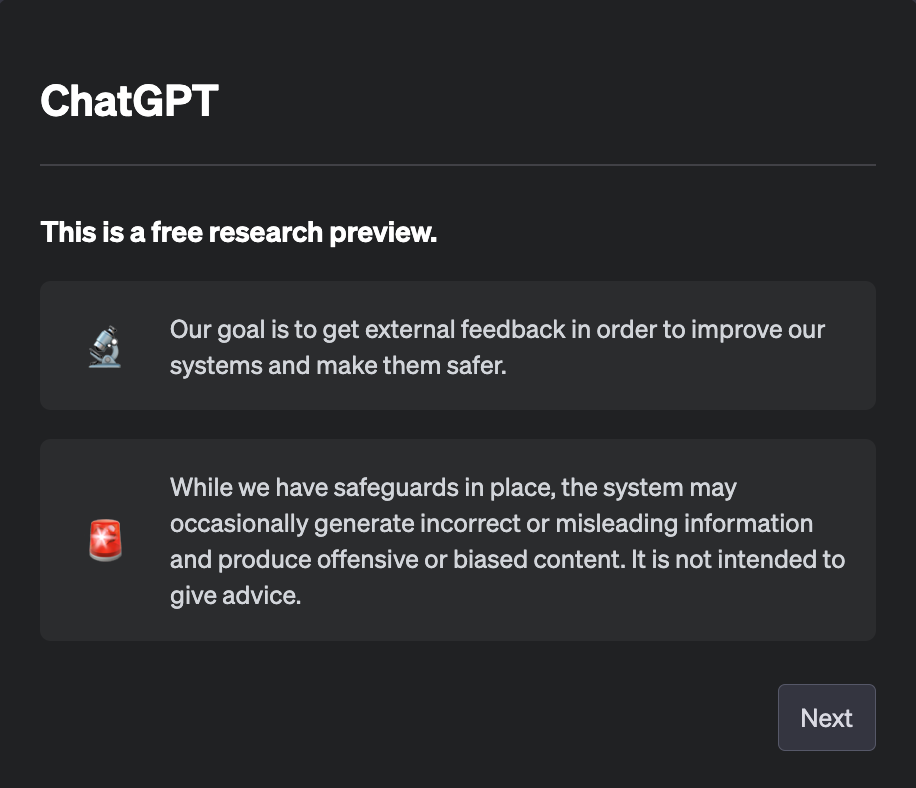


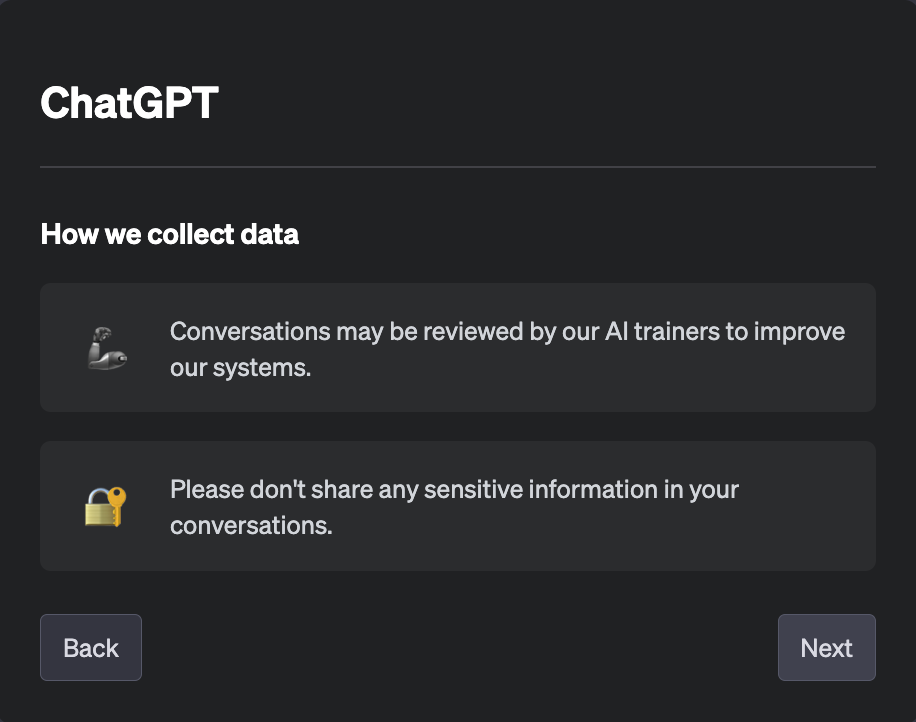


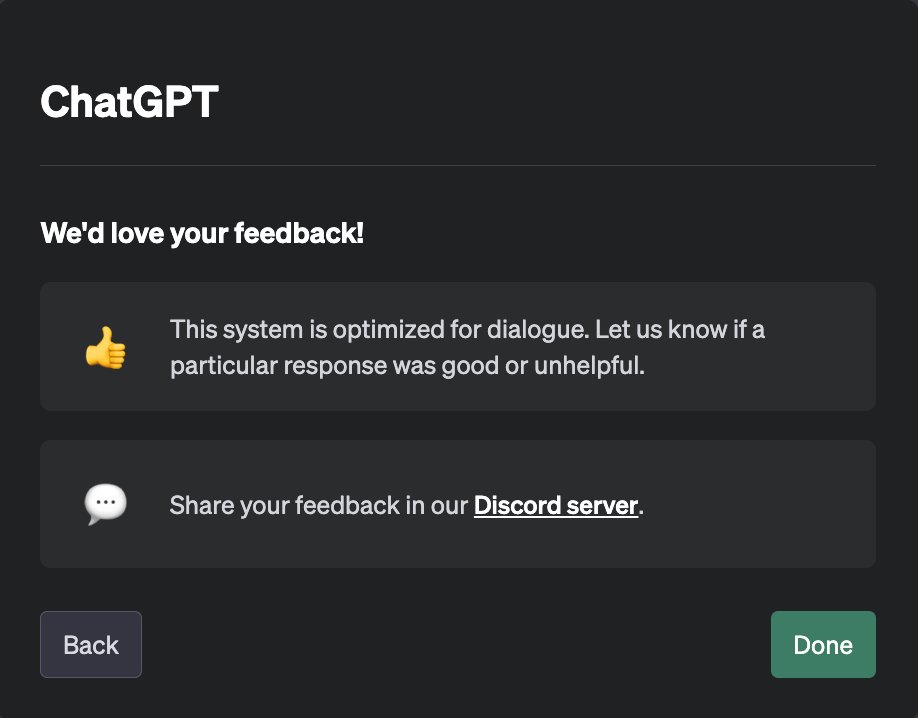

Supplement: Supplementary file 4 — ChatGPT Disclaimers (Screenshots), accessed 24 April 2023 (DOCX 243 kb) [file 590_2023_3742_MOESM4_ESM.docx]
