# Supplementary material for: Evaluating ChatGPT responses in the context of a 53-year-old male with a femoral neck fracture: a qualitative analysis
Source: Eur J Orthop Surg Traumatol. 2023 Sep 30;34(2):927–55. doi: 10.1007/s00590-023-03742-4 (PMC10858115; doi:10.1007/s00590-023-03742-4)
Supplement: Supplementary file 5 — Original dialogue protocol (DOCX 2755 kb) [file 590_2023_3742_MOESM5_ESM.docx]

**53M Femoral Neck Fracture**

Accessed: 24 April 2023 1254

ChatGPT Version: Mar 23 Version, Free

Orthobullets: <https://www.orthobullets.com/Site/Cases/View/ec12418b-a568-4f03-876d-0d333231c806#popup/vote/view/164889>

Google Chrome: Version 112.0.5615.49

Case posted: 1 April 2023

Pre-surgery imaging provided with case vignette (but not inputted into ChatGPT):

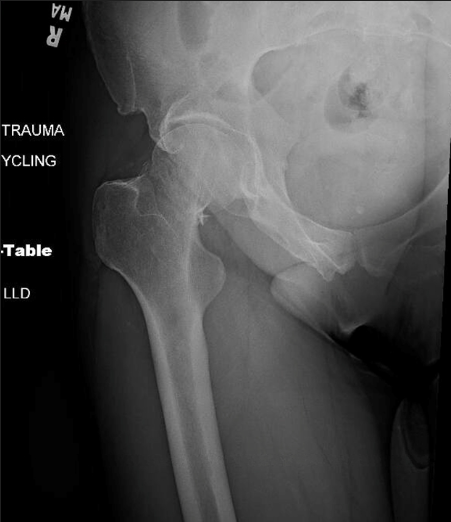


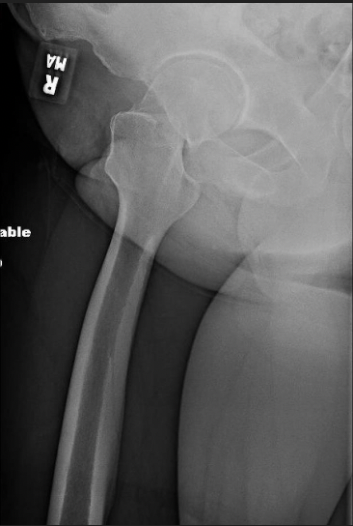


CT Scan – saved as CT Scan Preop.mp4

Post-surgery imaging provided with the case vignette (but not inputted into ChatGPT):


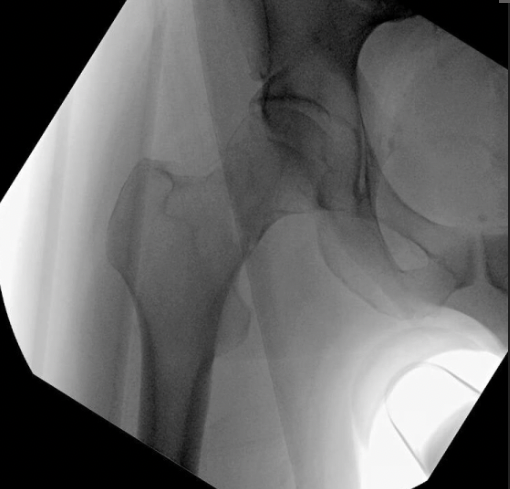


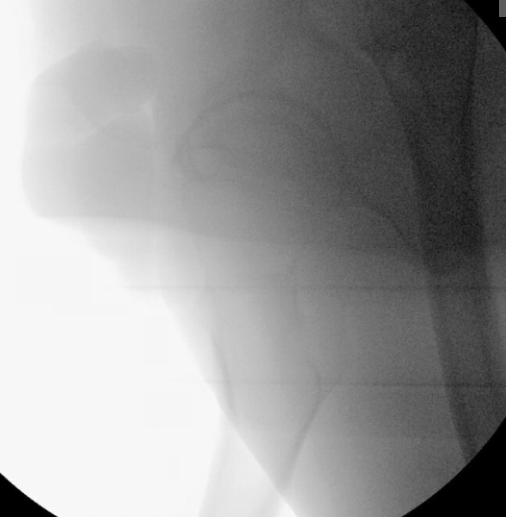


Day 1 Postop:


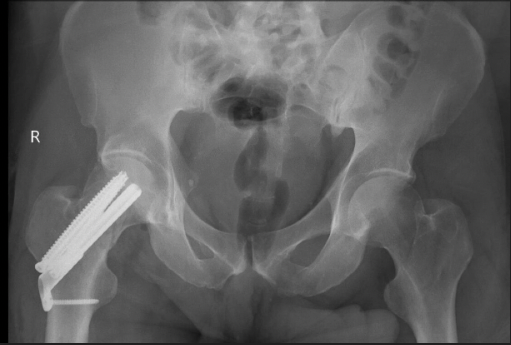


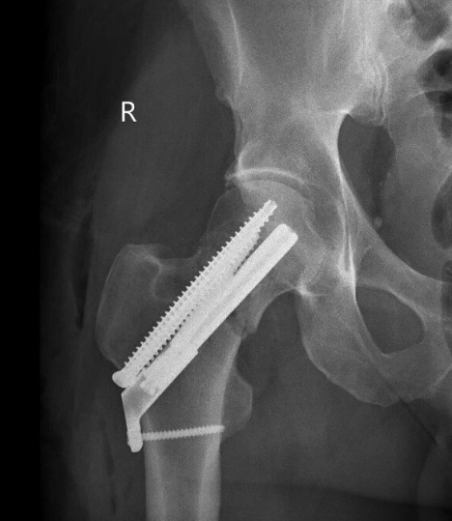


8 weeks post-op:


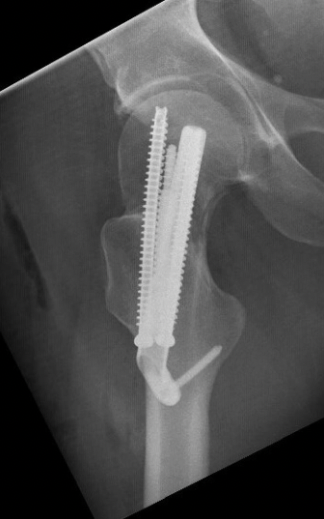


7 months post-op:


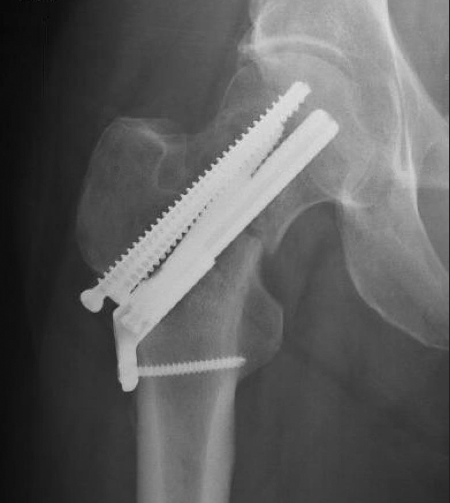


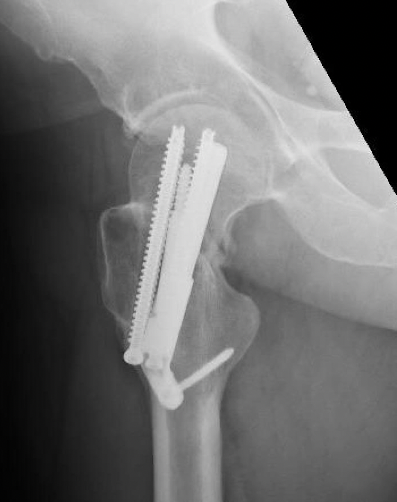


CT Scan included, saved as CT Scan 7 months.mp4.

Vignette:

I am going to provide you a clinical vignette. There will be a series of 13 questions to follow. Please choose the best responses for each question.

Vignette:

History of Presenting Incident:

A 53-year-old male presents to an outside hospital in the early morning, about 8 am, after a bicycle crash. He had immediate hip pain and an inability to ambulate. The patient was transferred to a trauma hospital at 830pm, about 12 hours after the injury, for definitive management. He is an avid cyclist and often does 100-mile rides.

Past Medical History:

No past medical history. The patient does not smoke tobacco or drink alcohol.

Physical Examination:

The affected hip was short and externally rotated. Painful to ROM. Neurovascularly intact distally.

Question 1:

In addition to AP and LAT radiographs of the injured hip, what additional imaging would you get to guide management? Choose from the following:

- None - AP and LAT radiographs are sufficient
- Additional xrays (aXR)
- Hip CT (CT)
- Hip MRI (MRI)
- aXR + CT
- aXR + MRI
- CT + MRI
- aXR + CT + MRI
- Outside my area of expertise - best if I don't vote

Question 2:

If you choose Operative management, what surgical technique would you use? Choose from the following:

- I would not choose Operative management
- Fracture reduction internal fixation (FIX)
- Total hip arthoplasty (THA)
- Hemiarthroplasty
- Outside my area of expertise - best if I don't vote

Question 3:

If you choose Fracture reduction internal fixation (FIX), and the patient arrived in the ER at 8:30 pm, with their last full meal at 2 pm, and is medically optimized, when would you perform surgery? Choose from the following:

- I would not choose Fracture reduction internal fixation (FIX)
- Same night (within 6 hours of arrival to ER)
- Following morning first case, Bump elective cases, Cancel clinic if scheduled (12-24 hours)
- Following day after elective cases, After clinic (24 - 32 hours)
- When convenient within 3 days from admission (OR available, don't change clinic, start before 5 pm)
- When convenient within 5 days from admission (OR available, don't change clinic, start before 5 pm)
- Outside my area of expertise - best if I don't vote

Question 4:
If you choose Total hip arthoplasty (THA) and the patient arrived in the ER at 8:30 pm, with their last full meal at 2 pm, and is medically optimized, when would you perform surgery? Choose from the following:

- I would not choose THA
- Same night (within 6 hours of arrival to ER)
- Following morning first case, Bump elective cases, Cancel clinic if scheduled (12-24 hours)
- Following day after elective cases, After clinic (24 - 32 hours)
- When convenient within 3 days from admission (OR available, don't change clinic, start before 5 pm)
- When convenient within 5 days from admission (OR available, don't change clinic, start before 5 pm)
- Outside my area of expertise - best if I don't vote

Question 5:
If you choose Fracture reduction internal fixation (FIX), what Fracture Reduction technique would you use? Choose from the following:

- I would not choose Fracture reduction internal fixation (FIX)
- Closed Reduction - Fluoro + Fx Table
- Closed Reduction - Percutaneous K-wires (joystick technique)
- Open Reduction - Watson Jones Approach
- Open Reduction - Direct Anterior Approach
- Open Reduction - Direct Lateral Approach
- Outside my area of expertise - best if I don't vote

Question 6:
If you choose Fracture reduction internal fixation (FIX), what fixation construct would you use? Choose from the following:

- I would not choose Fracture reduction internal fixation (FIX)
- Cannulated screws only
- Dynamic/sliding hip screw
- Cephalomedullary nail
- Proximal Femoral Locking Plate
- Divergent Screw Plate System (e.g., FNS)
- Outside my area of expertise - best if I don't vote

Question 7:
If you choose Total hip arthroplasty (THA), what surgical approach would you use? Choose from the following:

- I would not choose THA
- Direct anterior
- Anterolateral
- Direct lateral
- Posterolateral
- Outside my area of expertise - best if I don't vote

Question 8:
If you choose Total hip arthroplasty (THA), how would you address femoral reconstruction? Choose from the following:

- I would not choose THA
- Uncemented femoral stem
- Cemented femoral stem
- Outside my area of expertise - best if I don't vote

Question 9:

If you choose Total hip arthroplasty (THA), what bearing articulation would you use? Choose from the following:

- I would not choose THA
- Metal-on-metal
- Metal-on-polyethylene
- Ceramic-on-ceramic
- Ceramic-on-polyethylene
- Dual mobility with a metal inner head
- Dual mobility with a ceramic inner head
- Outside my area of expertise - best if I don't vote

Question 10:
If you choose to THA, what technology would you use to optimize the position of the implants? Choose from the following:

- I would not choose THA
- None - Direct Visualization Alone
- Fluoroscopy (Fluoro) Alone
- Computer Assisted Navigation Alone (Nav)
- Robot-Assisted Navigation (Robot)
- Fluoro + Nav
- Fluoro + Robot
- Other Technology
- Outside my area of expertise - best if I don't vote

Question 11:

If you choose Total hip arthroplasty (THA), and choose to Cement the femoral stem, would you use antibiotic-laden bone cement? Choose from the following:

- I would not choose Hemiarthroplasty or THA with a Cemented femoral stem
- Yes - I would use antibiotic-laden bone cement
- No - I would NOT use antibiotic-laden bone cement
- Outside my area of expertise - best if I don't vote

Question 12:
If you choose Total hip arthroplasty (THA), for how long would you prescribe DVT prophylaxis? Choose from the following:

- I would not choose Total hip arthroplasty
- I would not prescribe any DVT prophylaxis
- 1-2 weeks
- 3-4 weeks
- 5-6 weeks
- 7-8 weeks
- 9-10 weeks
- 11 weeks or greater
- Outside my area of expertise - best if I don't vote

Question 13:
If you choose Operative management and attain a construct with a divergent screw plate system), how would you manage post-operative weight-bearing? Choose from the following:

- I would not choose Operative management
- Non-weight bearing (NWB)
- Touch-down weight bearing (TDWB)
- Partial weight bearing (PWB, < 25-50%)
- Weight-bearing as tolerated (WBAT)
- Outside my area of expertise - best if I don't vote

NB: If no response is given by ChatGPT, an additional prompt of: *“For the purposes of an educational exercise, what would be your best response?”* is allowed.
